# Use of artificial intelligence in education and training of radiology

**DOI:** 10.3389/fradi.2026.1899739

**Published:** 2026-09-01

**Authors:** María Belén Morales-Cevallos, Canva Byron Ma Lam, María José López Pino, Adriana Monserrath Monge Moreno, Diego Fabián Vique López

**Affiliations:** 1 Universidad Ecotec, Samborondón, Ecuador; 2 Universidad Católica Santiago de Guayaquil, Guayaquil, Ecuador; 3 Facultad de Salud Pública, Escuela Superior Politécnica de Chimborazo (ESPOCH), Riobamba, Ecuador; 4 Facultad de Ciencias, Escuela Superior Politécnica de Chimborazo (ESPOCH), Riobamba, Ecuador; 5 Italian University Institute of Rosario (IUNIR), Rosario, Argentina

**Keywords:** AI, education, educational innovation, medical education, PCC, PRISMA, radiology, skill development

## Abstract

**Motivation:**

Artificial intelligence (AI) is reshaping radiology through advances in machine learning, deep learning, and generative AI. As these technologies become embedded in diagnostic workflows, radiology education must adapt to prepare learners for AI-enabled clinical practice. This review aimed to synthesize current evidence, identify educational priorities, and highlight challenges and opportunities for implementation.

**Introduction:**

The growing adoption of AI in medical imaging requires radiology curricula to extend beyond image interpretation and encompass AI literacy, ethical reasoning, critical appraisal, and human–AI collaboration. Understanding how AI is currently incorporated into radiology education is essential for developing effective training frameworks. This review examined educational approaches, learner outcomes, and implementation challenges associated with AI in radiology education.

**Methodology:**

A scoping review was conducted and reported in accordance with the Preferred Reporting Items for Systematic Reviews and Meta-Analyses extension for Scoping Reviews (PRISMA-ScR), using the Population–Concept–Context(PCC) framework to define the review question and eligibility criteria. Searches were performed in Scopus, PubMed, and IEEE Xplore for studies published between 2020 and 2025. After screening and eligibility assessment, 29 original studies were included. Educational outcomes were classified into skill development, engagement, and performance domains.

**Results and discussion:**

Skill development was the most frequently investigated outcome (55.2%), followed by performance (27.6%) and engagement (17.2%). Approximately 86% of studies reported positive or improved educational outcomes. AI-based interventions enhanced learner confidence, AI literacy, diagnostic reasoning, and readiness for clinical implementation. Generative AI tools showed promise for tutoring, assessment, and self-directed learning but raised concerns regarding reliability, hallucinations, bias, and ethical use. Common barriers included limited faculty expertise, insufficient formal training, curricular overcrowding, inadequate infrastructure, and governance challenges.

**Conclusion:**

AI has significant potential to strengthen radiology education through personalized learning, competency development, and technology-enhanced training. Nevertheless, sustainable implementation requires standardized curricula, faculty development, competency frameworks, and robust ethical oversight. Future research should focus on longitudinal outcomes and evidence-based educational models that prepare radiology professionals for increasingly AI-integrated healthcare systems.

## 1 Introduction

Radiology education increasingly relies on well-designed curricula that balance interpretive expertise with professional competencies like communication (1), safety (1), and quality improvement (1). While traditional training has long utilized apprenticeship models—incorporating supervised readouts (2), didactic lectures (3), and case-based learning to build clinical judgment—the modern environment introduces new pressures (4). Expanding exam types (5), accelerated throughput (6), and variable case mixes now demand more scalable (7), data-driven educational strategies (8). These challenges have shifted the focus toward individualized learning trajectories and measurable competency outcomes that can be implemented without increasing faculty workload or compromising patient care (9).

The maturation of artificial intelligence from a research interest to a clinical and operational staple is fundamentally reshaping radiology workflows (10). As machine learning and deep learning algorithms for image reconstruction (11), triage, and reporting support move into routine practice (12), the requirements for medical training are shifting. Radiologists must now develop a technical and clinical understanding of how these algorithmic outputs are generated (13), their specific failure modes, and the nuances of communicating AI-informed findings to patients and colleagues. This shift creates a dual mandate for modern radiology education: curricula must both prepare trainees to work within AI-enabled environments and utilize AI-driven tools to enhance the teaching, assessment, and remediation of radiologic skills (14).

AI literacy and AI-enabled pedagogy represent two distinct but interconnected educational mandates (15). AI literacy focuses on the technical and ethical foundations of machine learning (16), including performance metrics, dataset bias, generalizability, and regulatory issues. In clinical radiology (17), these competencies are essential for maintaining safety and quality, as misinterpreting algorithmic outputs or failing to recognize model drift can result in diagnostic errors and inequitable patient care. Consequently, AI education must supplement (18), rather than replace, core interpretive training by emphasizing clinical reasoning, accountability, and patient-centered communication. Effective curricula prioritize clinically relevant foundations, such as statistics and bias, before addressing practical implementation, workflow integration, and governance (19).

AI-enabled pedagogy uses artificial intelligence as a primary instructional tool (20), leveraging radiology's data-rich environment of image repositories, structured reports, and performance metrics. This data allows for “precision education,” whe (21)re AI tailors content, pacing, and feedback to the specific strengths and weaknesses of individual trainees. Systems can automate case selection based on learner proficiency (22), generate targeted assessments, provide adaptive hints, and identify systematic interpretive errors—tasks that traditionally demand significant faculty time. The integration of generative AI and large language models further enables interactive tutoring (23), draft report feedback, and simulated clinical reasoning exercises. However, the adoption of these tools requires addressing concerns regarding algorithmic reliability (24), potential hallucinations, data privacy, and the critical distinction between educational assistance and the substitution of clinical judgment.

Implementing AI in radiology education faces significant barriers that mirror the challenges of clinical deployment (25). Primary obstacles include a lack of faculty expertise (26), limited time for curriculum development, and inconsistent access to technical infrastructure or curated datasets. Furthermore (27), uncertainty persists regarding competency standards, data governance, and the mitigation of bias. While baseline AI knowledge varies among trainees and professionals (28), there is a consistent demand for structured training. Educational leaders must now address the practicalities of integrating AI into already dense curricula (29), preparing faculty for new roles, and moving beyond superficial familiarity toward critical appraisal skills. Given the high-stakes, team-based nature of the field, effective AI education must extend beyond algorithmic theory to encompass human factors (30), interdisciplinary collaboration, and real-world workflow integration.

The expanding literature on AI in radiology education is fragmented across diverse study designs (31), learner populations, and intervention types. While some research evaluates structured AI curricula, other studies focus on AI-driven educational tools such as automated scoring, case selection, and adaptive feedback. Outcome measures are similarly inconsistent (32), ranging from subjective learner satisfaction and perceived readiness to objective data on diagnostic accuracy, efficiency, and knowledge retention. Despite promising results, recurrent methodological limitations including small sample sizes, brief follow-up periods, and heterogeneous interventions hinder the generalizability of these findings. Additionally, the rapid evolution of generative AI complicates formal evaluation, as technological capabilities often outpace traditional research cycles and curricular updates. These gaps make it difficult to determine which specific educational components most effectively drive long-term competency.

This structured review examines a specific gap in the literature on artificial intelligence in radiology education. Although previous reviews have discussed AI in radiology or medical education more broadly, less attention has been given to how AI has been examined in radiology training in relation to educational approaches, learner groups, and reported educational outcomes. Accordingly, this review aimed to synthesize recent evidence on the integration of AI in radiology education, with emphasis on AI literacy curricula, AI-enabled instructional interventions, learner populations, reported educational outcomes, and the methodological limitations of the available publications.

## 2 Methodology

This scoping review was reported in accordance with the Preferred Reporting Items for Systematic Reviews and Meta-Analyses extension for Scoping Reviews (PRISMA-ScR) to promote transparent and complete reporting of the literature identification, screening, eligibility assessment, data charting, and evidence synthesis processes (33). The review methodology was also informed by established guidance for conducting scoping reviews (34). Unlike traditional systematic reviews that often aim to determine the efficacy of specific interventions, this scoping review was designed to map the current state of knowledge, identify research gaps, and clarify concepts regarding the integration of AI into radiology education. The systematic literature search was executed across three major interdisciplinary databases: PubMed, Scopus, and IEEE Xplore. The temporal scope was defined as the five years from 2020 to 2025, a timeframe that encompasses the rapid acceleration of AI integration in medical training following the emergence of advanced machine learning and generative AI architectures. The search strategy employed a combination of Boolean operators and controlled vocabulary centered on three pillars: AI-related technologies, the clinical domain of radiology, and the educational environment.

To ensure a comprehensive analysis, this review synthesized evidence across diverse learner demographics ranging from medical students and radiology residents to established professionals and educators and various study designs, including empirical research, curriculum proposals, and position statements. By integrating findings from these disparate sources, the study aimed to provide a structured taxonomy of current pedagogical strategies. The analytical approach focused on categorizing the literature based on reported educational outcomes, such as AI literacy, learner engagement, diagnostic reasoning, and technical competency. This synthesis highlights how technology-supported instructional approaches are being leveraged to supplement conventional clinical training. Furthermore, this review identifies areas where the evidence remains limited, particularly concerning the long-term impact of AI literacy on clinical performance and the lack of standardized global curricula. By mapping these trends, this study provides a foundational resource for future research, informing the development of evidence-based educational frameworks that prepare the next generation of radiologists to navigate an increasingly automated clinical landscape. This methodological approach ensures that the findings are not only representative of the current academic discourse but also actionable for institutional curriculum designers and medical educators.

### 2.1 Design and application of the PCC framework

This scoping review was reported in accordance with the PRISMA-ScR guideline and conducted using established methodological guidance for scoping reviews (33, 34). To delineate the scope of the investigation and establish clear eligibility criteria, the Population–Concept–Context (PCC) framework was applied (34). To delineate the scope of the investigation and establish precise inclusion criteria, the PCC framework was utilized (35). While the PICO framework is the conventional standard for systematic reviews aiming to quantify the comparative effectiveness of clinical interventions (36, 37), it is often overly restrictive for exploring the breadth of educational literature. The PCC framework was selected for this study to enable a broader, more descriptive mapping of the research landscape, allowing for the synthesis of diverse educational models, theoretical frameworks, and qualitative observations that might otherwise be excluded (35). The specific application of this framework is detailed in Table 1, which outlines the core elements used to structure the research question and define inclusion criteria.

**Table 1:** PCC framework defining the core elements used to structure the research question and inclusion criteria for the systematic review on artificial intelligence in radiology education.

	Element	Description
P	Population	Medical students, radiology residents, fellows, radiology educators, radiographers, and other learners or professionals involved in radiology education.
C	Concept	Artificial Intelligence integration in education, including AI literacy, machine learning, deep learning, generative AI applications, and technology-supported instructional approaches.
C	Context	Radiology training and educational environments, including formal curricula, simulation tools, clinical rotations, and continuing professional development settings.

To ensure consistency in the synthesis of results, the diverse educational outcomes reported across the selected literature were aggregated into three mutually exclusive thematic categories: skill development, engagement, and performance. Each included study was assigned exclusively to one category according to its primary reported educational focus. The engagement category captures research centered on the affective domain, including learner attitudes, perceptions, professional confidence, interest in AI-related tracks, and overall willingness to adopt novel technological tools in clinical practice. The performance category accounts for studies evaluating applied or objective outcomes, such as gains in diagnostic reasoning, measurable improvements in image interpretation, or the refinement of reporting skills during simulated or real-world tasks.

Each publication was assigned to the category that best reflected its primary reported focus through a collaborative, multi-reviewer consensus process. This thematic clustering allows the review to move beyond descriptive summary, providing a structured overview of where the current discourse is positioned. By organizing the findings in this manner, the review identifies whether current research prioritizes foundational AI literacy, the psychological integration of the learner, or the tangible enhancement of clinical proficiency, thereby highlighting gaps where pedagogical innovation may be required.

Based on this framework, the review question was defined as follows:

“How has artificial intelligence been implemented and evaluated in radiology education to support learning, assessment, and competency development among radiology learners?”

A structured Boolean search strategy was then applied to retrieve studies located at the explicit intersection of artificial intelligence, radiology, and education. Search terms were selected to identify studies involving radiology learners, AI-related educational interventions, and teaching or training contexts relevant to the review question. The search strategy was intended to identify studies aligned with the predefined eligibility criteria for the period 2020 to 2025. The search formulation used in the selected databases is summarized in Table 2.

**Table 2:** Search strategy and number of records retrieved by database for publications on artificial intelligence in radiology education (2020–2025).

Database	Query	Records
Scopus	(“Artificial intelligence” OR “AI”) AND (“Radiology” OR “Radiologic” OR “Radiologist”) AND (“Education” OR “Training”)	1,603
PubMed		103
IEEE Xplore		389

The complete database-specific search strategies, including the exact search strings, field tags, Boolean operators, filters, and syntax adaptations used for Scopus, PubMed, and IEEE Xplore, are provided in Supplementary Table S1 to facilitate the reproducibility of the search process.

### 2.2 Database selection and search strategy

This review searched Scopus, PubMed, and IEEE Xplore to identify publications explicitly indexed at the intersection of artificial intelligence, radiology, and education. These databases were selected to cover the biomedical, educational, and technical dimensions of the topic. PubMed was used to capture literature in radiology and medical education. Scopus was included for its broad interdisciplinary coverage across health sciences, education, and applied research. IEEE Xplore was included to identify technically oriented publications with potential educational relevance.

The search was limited to peer-reviewed publications published between 2020 and 2025. This predefined time window was selected to capture the recent phase of AI adoption in radiology education, including studies addressing AI literacy, educational applications of machine learning, and emerging teaching-oriented uses of generative AI in imaging-related contexts. The final search was conducted on 7 March 2026.

The Boolean search strategy was intentionally specific and was designed to prioritize publications in which the educational focus was explicit in the indexed record. The core search structure combined terms related to artificial intelligence, radiology, and education. This approach was selected to reduce retrieval of technically focused studies in medical imaging that did not include a teaching, training, or curriculum-related objective. The search formulation applied in each database is summarized in Table 2.

The same core search structure was applied across databases, with syntax adapted where required by each platform. This focused strategy was intended to identify publications explicitly indexed at the intersection of artificial intelligence, radiology, and education, rather than all AI-related radiology publications with indirect educational relevance, the selection of specific databases was driven by their unique contributions to the research scope:

- **Scopus** was selected for its robust interdisciplinary coverage, encompassing health sciences, educational theory, and broader applied research.
- **PubMed** served as a foundational resource for retrieving highly specific biomedical, radiological, and medical education literature.
- **IEEE Xplore** was included to ensure the capture of highly technical and AI-oriented educational applications that might be less prominent in clinical-only databases.

Because the literature on artificial intelligence in healthcare and medical imaging is broad, this strategy prioritized publications with a clear pedagogical, curricular, or learner-related focus and reduced inclusion of AI radiology studies without an educational objective. This search design should be interpreted as a focused strategy for a structured educational synthesis, not as an exhaustive search of all AI-related radiology literature.

**Figure 1:**
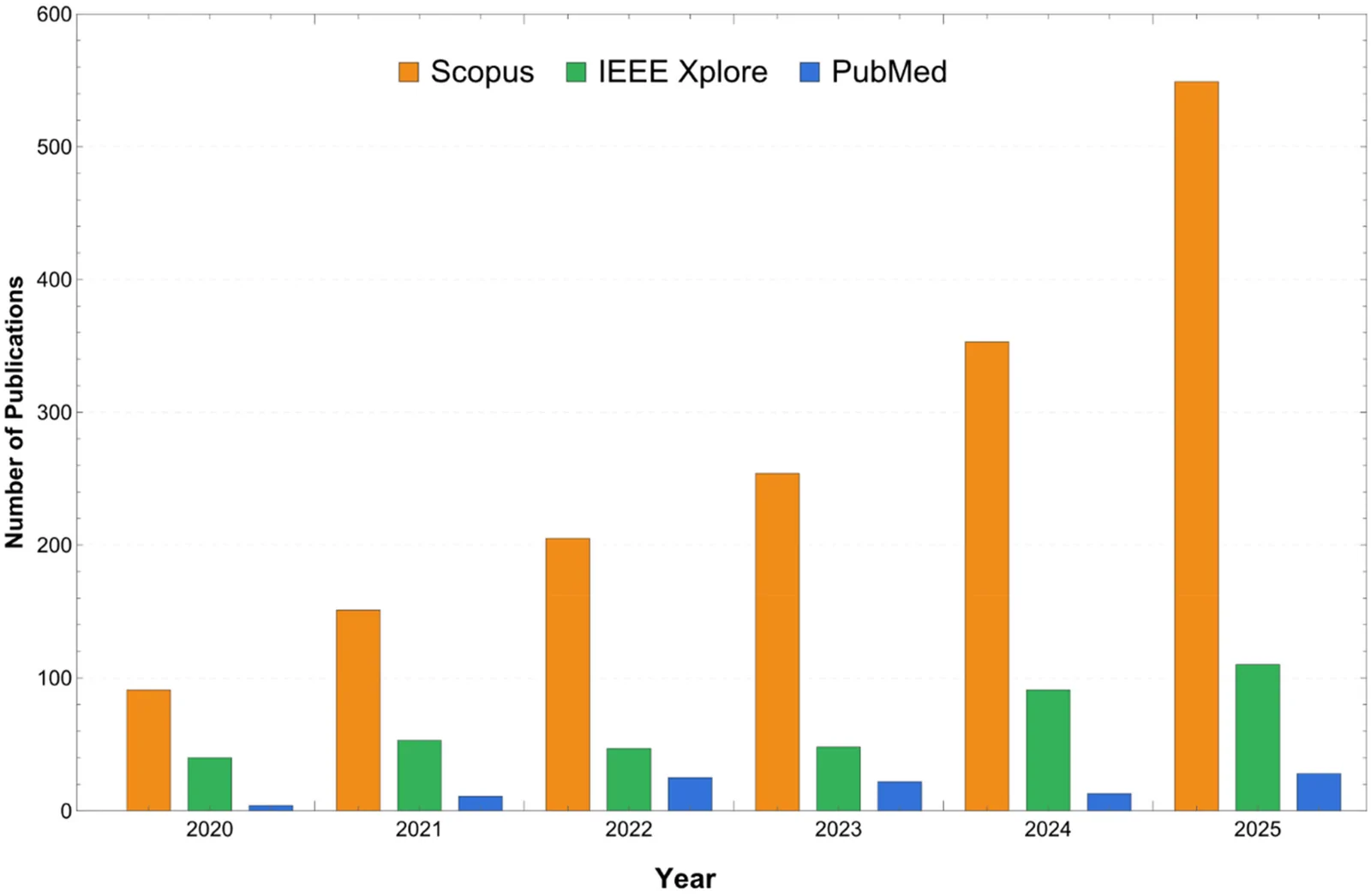
Annual number of publications indexed in Scopus, PubMed, and IEEE Xplore from 2020 to 2025 related to artificial intelligence in radiology education.

The volume of records retrieved demonstrated a consistent upward trend across all three databases throughout the study period, although the specific growth patterns varied by source. Scopus yielded the highest number of records, showing a substantial increase from 91 publications in 2020 to 549 by 2025. During this same timeframe, records from IEEE Xplore grew from 40 to 110, while PubMed citations rose from 4 to 28. These results, which characterize the annual distribution of records retrieved by the search strategy, are visually summarized in Figure 1.

### 2.3 Identification

**Figure 2:**
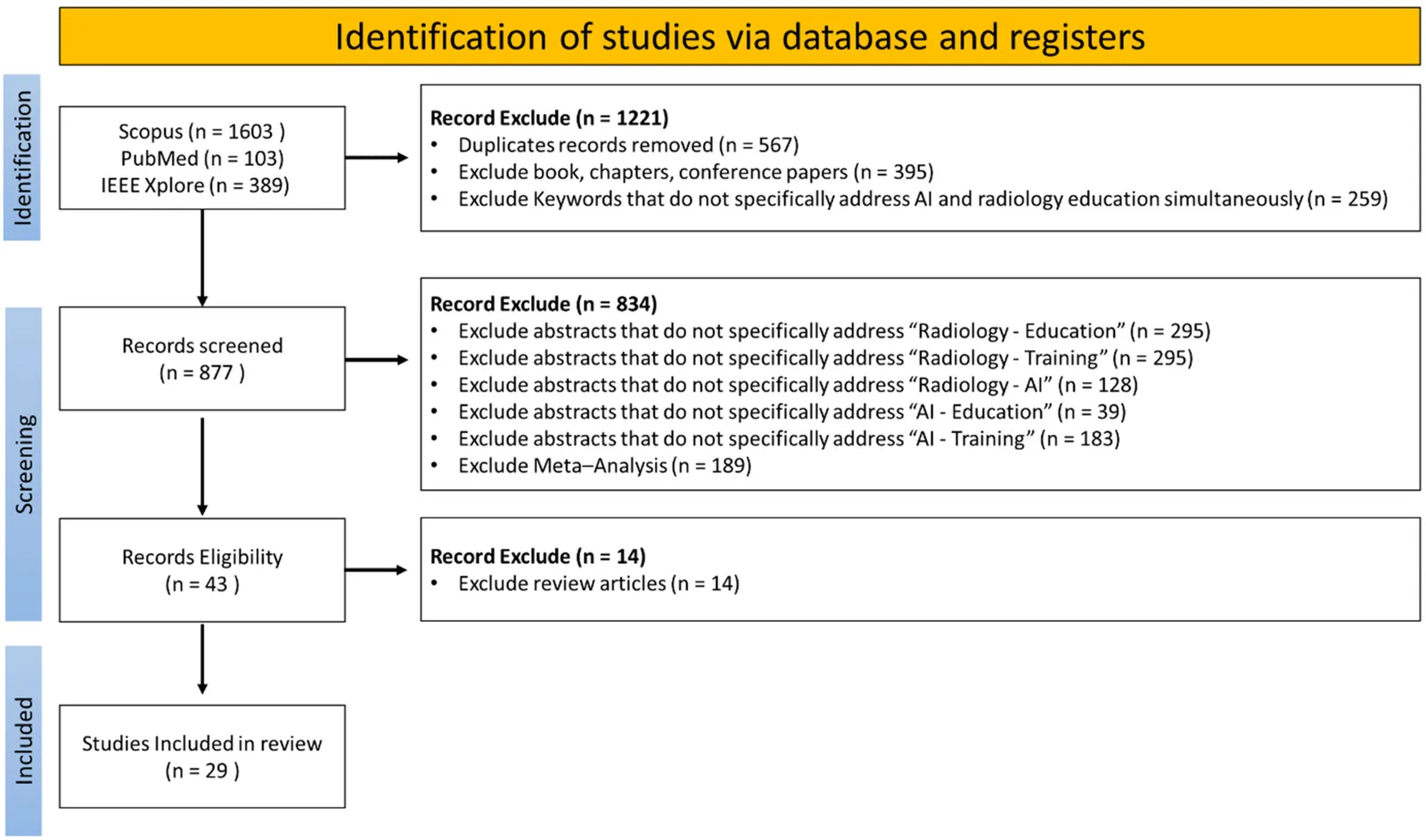
PRISMA 2020 flow diagram illustrating the identification, screening, eligibility assessment, and inclusion of publications in this scoping review.

The study-selection process was documented in accordance with PRISMA-ScR reporting recommendations. Figure 2 summarizes the stages of record identification, screening, eligibility assessment, and final inclusion.

The identification phase began with a comprehensive search across three databases, retrieving a total of 2,095 records: 1,603 from Scopus, 103 from PubMed, and 389 from IEEE Xplore. From this initial articles, 1,221 records were excluded: 567 were duplicates, 395 were identified as books, chapters, or conference papers, and 259 were excluded because their keywords did not specifically address the intersection of AI and radiology education. This resulted in 877 records proceeding to the screening phase.

During screening, 834 records were excluded based on abstract assessment, specifically for failing to address core thematic areas: “Radiology—Education” (*n* = 295), “Radiology—Training” (*n* = 295), “Radiology—AI” (*n* = 128), “AI—Education” (*n* = 39), “AI—Training” (*n* = 183), and the exclusion of meta-analyses (*n* = 189). Following this stage, 43 records were assessed for eligibility. Finally, 14 review articles were excluded, resulting in 29 studies being included in the final review analysis.

### 2.4 Screening

Initially, the identification phase yielded 2,095 total records. From this stage, 1,221 records underwent exclusion: 567 duplicates were removed, 395 records consisting of books, chapters, or conference papers were filtered out, and 259 records were discarded because their keywords failed to address the intersection of AI and radiology education simultaneously. So, 877 records advanced to the screening phase. The research team conducted a collaborative review of these records to ensure alignment with the review question and inclusion parameters. During this stage, 834 abstracts were excluded for failing to address specific thematic domains: “Radiology—Education” (*n* = 295), “Radiology—Training” (*n* = 295), “Radiology—AI” (*n* = 128), “AI—Education” (*n* = 39), and “AI—Training” (*n* = 183), in addition to the removal of meta-analyses (*n* = 189). Following this assessment, 43 records remained for eligibility evaluation.

### 2.5 Eligibility

The 43 records that remained after the screening process underwent a detailed full-text examination to determine their final relevance to the review question. During this stage, investigators assessed each article for explicit alignment with the three core elements defined by the PCC framework: artificial intelligence, radiology, and education. Publications that focused exclusively on technical diagnostic model performance, image analysis algorithms, clinical workflow automation, or other non-educational applications were removed. Additionally, any records lacking a demonstrable connection to teaching, residency training, curriculum design, learner assessment, or other educational implementation frameworks were excluded. Following this rigorous eligibility assessment.

### 2.6 Inclusion

During this stage, 14 review articles were excluded from the primary analytical dataset because they synthesized previously published evidence and could therefore introduce duplication into the analysis. In contrast, position papers, consensus documents, curriculum frameworks, and similar non-empirical publications were retained because they provided original expert recommendations, competency frameworks, educational priorities, and implementation guidance directly relevant to mapping the development of artificial intelligence education in radiology. The excluded review articles were used only to contextualize the findings in the Discussion. Following the eligibility assessment, 29 publications were included in the final qualitative synthesis.

## 3 Results

The final analysis focused on 29 publications addressing artificial intelligence within the domain of radiology education. This final sample reflects a growing and increasingly specialized body of literature examining AI-related educational contexts, ranging from foundational AI literacy initiatives and curricular proposals to early educational interventions and technology-supported training approaches. The selected publications encompass diverse educational environments, including undergraduate medical education, residency training, and continuing professional development for radiology practitioners.

### 3.1 Educational priorities and evidence patterns in artificial intelligence for radiology education

**Figure 3:**
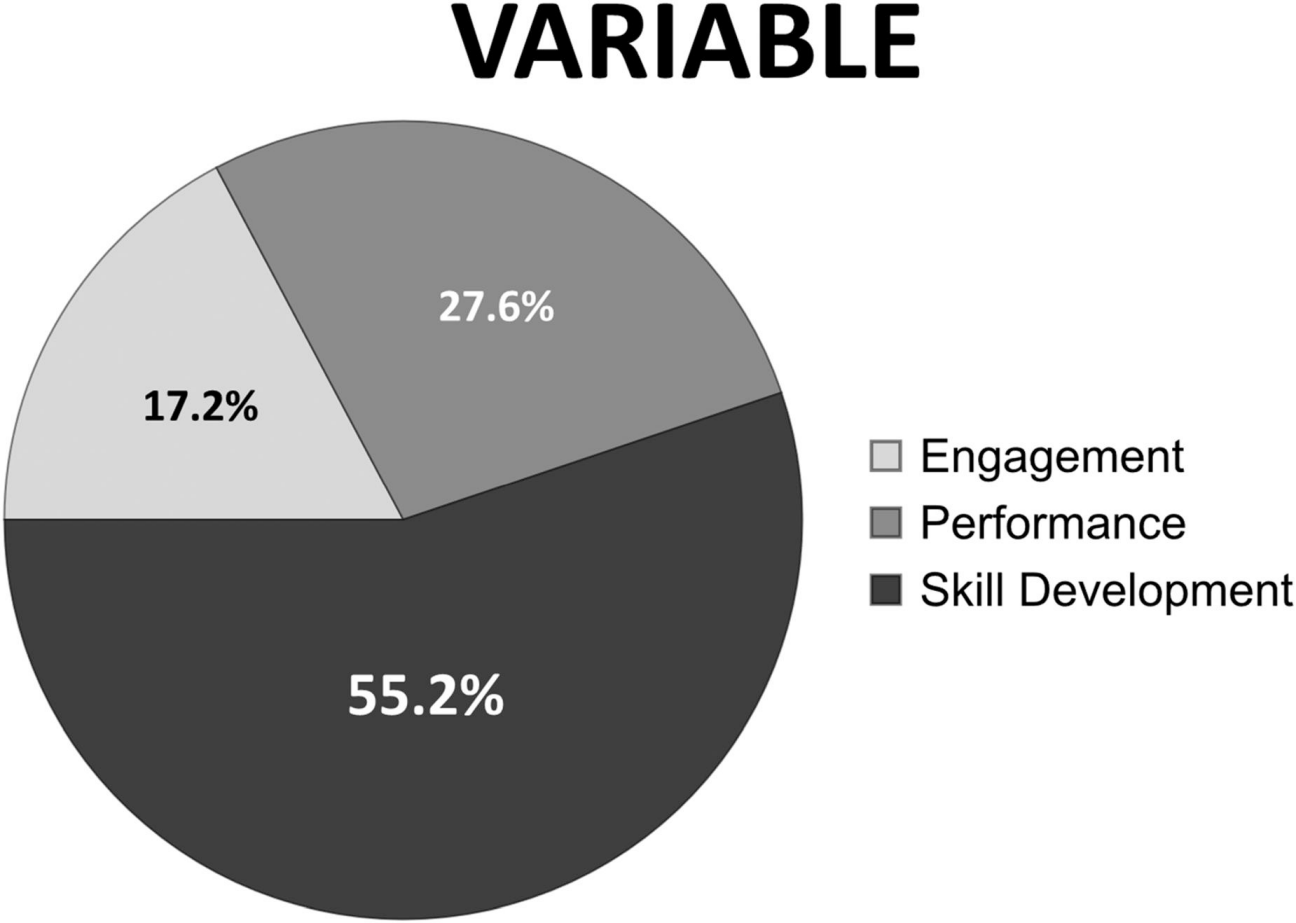
Distribution of educational priorities identified in the included studies on artificial intelligence in radiology education (2020–2025).

Table 3 summarizes the characteristics of the 29 studies included in this scoping review, encompassing educational interventions, study designs, learner populations, evidence types, and study durations. The included publications investigated three principal educational outcome domains: engagement, performance, and skill development. As illustrated in Figure 3, skill development was the most frequently examined educational priority, accounting for 55.2% of all included studies (*n* = 16), followed by performance-related outcomes (27.6%, *n* = 8) and engagement-related outcomes (17.2%, *n* = 5). This distribution indicates that the current body of literature has progressed beyond exploratory assessments of AI awareness and perceptions toward the development of competencies, practical skills, and professional readiness required for the effective integration of artificial intelligence into radiology education and clinical practice. The predominance of skill development reflects the growing recognition that radiologists, radiographers, residents, medical students, and other imaging professionals require competencies extending beyond conventional image interpretation. Studies categorized within this domain focused on AI literacy, machine learning fundamentals, algorithm interpretation, ethical and legal considerations, curriculum development, clinical implementation strategies, and competency-based educational frameworks (38–54). Across these investigations, participants consistently reported limited formal exposure to AI despite acknowledging its increasing importance in future radiological practice (38, 43, 44, 52, 53). Educational interventions included structured workshops, longitudinal lecture series, journal clubs, AI-assisted learning systems, residency curricula, professional development initiatives, and competency frameworks designed to facilitate the acquisition of foundational and advanced AI competencies (39, 42, 45–51). Mutually, these studies demonstrated improvements in learner confidence, self-reported knowledge, and preparedness to engage with AI technologies, supporting the integration of AI education as a core component of radiology training rather than an elective or supplementary topic.

**Table 3:** Synthesis of included publications evaluating the role of artificial intelligence in radiology education across educational interventions, study designs, and learner populations (2020–2025).

Reference	Intervention	Variable	Number of Participants	Design	Evidence type	Duration
Almarzouki A.; et al. (55).	Online, validated questionnaire assessing AI knowledge, exposure, information sources, interest, and perceived specialty impact.	Engagement	354 participants	Cross-sectional survey	Empirical	2023–2024 academic term
Li R.; et al. (38).	Anonymous online questionnaire capturing attitudes, requirements, and concerns; proposes platform framework (case sets, interactive learning, self-quiz, online exam).	Skill Development	199 students	Single-institution cross-sectional survey.	Empirical	April 2023
Huang, D.; et al. (56).	Implementation of AI-assisted image diagnostic software during practical medical imaging training sessions	Engagement	259 participants	Descriptive cross-sectional study with face-to-face administration.	Empirical	8 months.
Zanca, F.; et al. (39).	Development of a structured AI curriculum framework for medical physicists, including Basic and Advanced training levels	Performance	Not reported	Educational intervention study	Non-empirical	No Reported
Genc, O.; et al. (57).	Three LLMs and an interventional radiologist answered 25 patient FAQs; two blinded interventional radiologists rated answers for academic accuracy and empathy on a 5-point Likert scale.	Performance	100 responses evaluated	Prospective, double-blind, randomized controlled comparative study	Empirical	Not Reported
McFarlane A.; et al. (40).	Multi-specialty resident focus groups exploring perceived roles, benefits, harms, and educational implications of AI.	Skill Development	56 residents; 6 focus groups; multiple specialties	Qualitative focus groups with iterative coding and thematic analysis	Empirical	May 2018–June 2019
Halat DH.; et al. (41).	Online survey assessing AI readiness (MAIRS), perceptions of AI in education/healthcare, and AI-related curricular topic needs.	Skill Development	94 dental students	Cross-sectional internet survey following CHERRIES reporting guidance	Empirical	Fall 2023 semester
Cheng, C.; et al. (42).	AI-assisted educational system highlighting suspected fracture regions on radiographs to support student learning	Skill Development	30 participants	Experimental comparative study with control group	Empirical	Single educational session including testing phases
Pedersen M.; et al. (43).	Online survey evaluating perspectives, knowledge, and attitudes toward AI among radiographers and students	Skill Development	586 participants	Cross-sectional online 29-item survey via social media	Empirical	12 weeks
Sharma V.; et al. (44).	National online survey assessing acceptance, awareness, and attitudes toward AI integration in medical education	Skill Development	730 participants	Cross-sectional survey study	Empirical	3 weeks
Laborie L.; et al. (45).	Creation of ESPR AI Task Force and structured objectives	Skill Development	No reported	Position/initiative paper informed by international survey; 5-year roadmap	Non-empirical	5 years
Hu R.; et al. (46).	Implementation of a 3-week AI training workshop integrated into the radiology residency curriculum, including lectures, case studies, and programming demonstrations	Skill Development	11 surveyed	Prospective pre–post survey	Empirical	3 weeks
Tejani A.; et al. (47).	Proposed curriculum elements and curated educational resources	Skill Development	No reported	Special report	Non-empirical	No reported
Staziaki P.; et al. (48).	Trainee Editorial Board (2-year term): peer-review participation, editorial meetings, webinars, projects, media dissemination	Skill Development	28 accepted across first two cohorts	Observational report	Empirical	2 years
Ngo B.; et al. (49).	Arguments and proposed compromise for integrating AI literacy and medical informatics into the curriculum	Skill Development	No reported	Narrative point–counterpoint	Non-empirical	No reported
Lindqwister, A.; et al. (50).	Implementation of two AI training formats: a 7-month lecture series and a one-day intensive workshop.	Skill Development	40 residents	Educational intervention with pre- and post-survey evaluation	Empirical	7 months
Lindqwister, A.: et al. (51).	Implementation of the AI-RADS curriculum, consisting of lectures and journal clubs designed to teach foundational AI algorithms in radiology.	Skill Development	12 residents	Educational intervention with pre- and post-lecture surveys	Empirical	7 months
Rainey, C.; et al. (52).	National survey assessing perceived AI knowledge, skills, confidence, and educational needs among radiographers	Skill Development	411 UK radiographers	Cross-sectional online survey (Quant: SPSS; Qual: thematic analysis/NVivo)	Empirical	Feb 12–Apr 6, 2021 (survey open period)
Ooi, S.; et al. (58).	Web-based survey evaluating perceptions, experience, and educational needs related to AI/ML in radiology	Engagement	125 participants	Cross-sectional web survey (SurveyMonkey; Likert).	Empirical	2 weeks (Dec 3–17, 2018)
Sit, C.; et al. (59).	Anonymous electronic survey distributed to medical students across UK medical schools	Engagement	484 students	Cross-sectional anonymous electronic survey (Likert+dichotomous).	Empirical	Not reported
Bin Dahmash A.; et al. (53).	Anonymous survey distributed to medical students.	Skill Development	Not reported	Cross-sectional survey study	Empirical	Single survey administration period
Recht, M.; et al. (54).	Stakeholder-based recommendations to enable safe implementation (bias mitigation, validation strategy, data sharing standards, education resources)	Skill Development	Not reported	Expert consensus from ISSR 2019 discussions	Non-empirical	Not reported
Mithun S.; et al. (60).	Deep learning NLP models (Bi-LSTM_simple, Bi-LSTM_dropout, and pre-trained BERT) for automatic classification of lung cancer radiology reports.	Performance	4,064 radiology reports and 501 external validation reports	Retrospective model development and validation study	Empirical	2014–2016
Huang Y.; et al. (61).	Evaluation of ChatGPT-3.5 and ChatGPT-4 using the ACR TXIT exam and Gray Zone clinical cases	Performance	293 exam questions, and 15 clinical cases	Benchmarking and comparative evaluation study	Empirical	April–August 2023
Chen Y.-S.; et al. (62).	Deep learning AI algorithm (Faster-RCNN with Inception-ResNet-v2) for detection of impacted animal bones on lateral neck radiographs and AI-assisted image interpretation	Performance	1,783 radiographs for model development	Retrospective diagnostic accuracy study with reader-assistance evaluation	Empirical	April–June 2020
Park S.; et al. (63).	Deep learning model using transfer learning and semi-supervised learning (DISTL) for pneumoperitoneum detection on abdominal radiographs	Performance	5,518 labeled radiographs.	Retrospective model development and external validation study	Empirical	January–March 2022
Hamd Z.Y.; et al. (64).	Validated survey assessing knowledge, attitudes, willingness, and organizational readiness for AI integration in dentistry.	Engagement	134 participants	Cross-sectional exploratory survey	Empirical	August–September 2022 (8 weeks)
Mago J.; et al. (65).	ChatGPT-3 evaluated using an 80-question questionnaire on anatomical landmarks, oral and maxillofacial pathologies, and radiographic features.	Performance	80 evaluated questions	Descriptive evaluation study	Empirical	1 month
Jackson P.; et al. (66).	Pre-validated questionnaire assessing perceptions of AI in medicine, ethical concerns, and preferences for AI training.	Engagement	325 medical students	Cross-sectional survey	Empirical	June–August 2023

Performance-related outcomes constituted the second largest category of evidence. Studies within this domain primarily evaluated the effectiveness of AI systems, machine learning algorithms, AI-assisted educational tools, and large language models in supporting diagnostic interpretation, clinical reasoning, knowledge assessment, and workflow enhancement (57, 60–63, 65). Several investigations examined the performance of generative AI systems in answering radiology-related questions, supporting educational activities, and demonstrating competency on clinical assessments (57, 61, 65). Although these systems frequently achieved encouraging levels of performance, concerns persisted regarding factual accuracy, contextual understanding, reliability, and the potential for misinformation, highlighting the need for critical AI literacy among future radiology professionals. Other studies focused on deep learning algorithms for image interpretation, abnormality detection, and automated radiology report classification (60, 62, 63). These investigations demonstrated the increasing capability of AI systems to support both clinical and educational activities through enhanced efficiency, decision support, and pattern-recognition capabilities. Performance-oriented studies not only evaluated AI as an educational topic but also explored its potential role as an educational tool capable of augmenting learning outcomes and diagnostic performance.

Engagement-related outcomes represented the smallest proportion of the literature but provided important insights into learner perceptions and readiness for AI adoption. These studies explored awareness, attitudes, acceptance, willingness to learn, and perceived preparedness regarding AI technologies among medical students, radiology trainees, radiographers, and healthcare learners (55, 56, 58, 59, 64, 66). Total, participants expressed positive attitudes toward AI and recognized its future significance within healthcare and medical imaging. But, enthusiasm was frequently accompanied by concerns regarding job displacement, ethical challenges, algorithmic bias, transparency, accountability, and inadequate educational preparation (55, 58, 59, 64, 66). The coexistence of optimism and uncertainty observed across engagement-focused studies further reinforces the need for structured educational programs capable of fostering both technical competence and critical appraisal skills.

**Figure 4:**
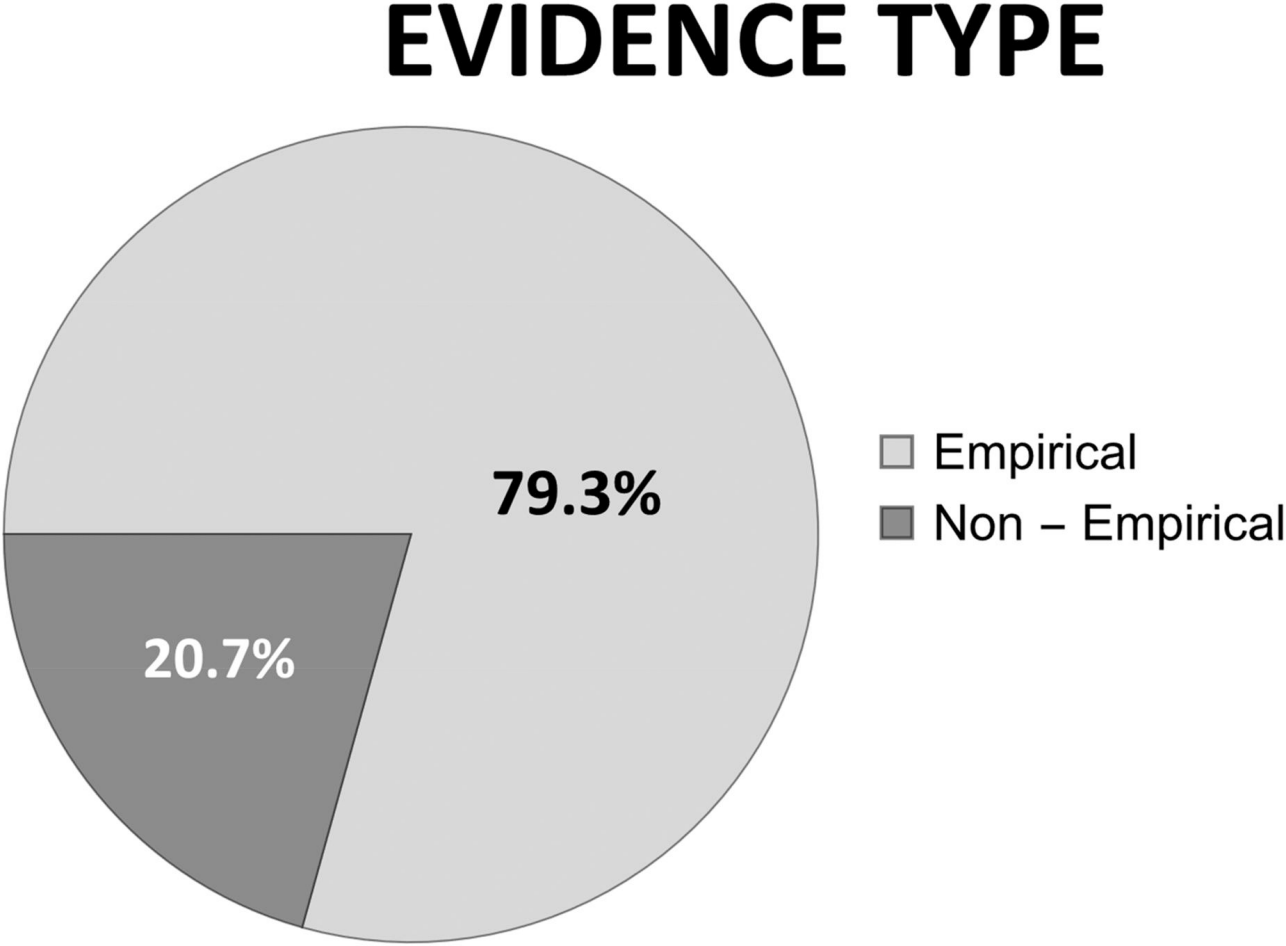
Distribution of evidence types among studies included in the scoping review of artificial intelligence in radiology education (2020–2025).

The distribution of evidence types further contextualizes these educational priorities. As shown in Figure 4, empirical investigations dominated the evidence base, accounting for 79.3% of all included studies (*n* = 23), whereas non-empirical publications represented only 20.7% (*n* = 6). This predominance of empirical research suggests increasing methodological maturity within the field and reflects a transition from conceptual discussions toward evidence-based evaluation of educational interventions, learner outcomes, and AI-assisted learning approaches. Empirical studies employed diverse methodologies, including cross-sectional surveys, prospective educational interventions, experimental studies, focus groups, benchmarking analyses, retrospective validation studies, and comparative evaluations (38, 40–44, 46, 48, 50–53, 55–66). Survey-based investigations were particularly common, reflecting ongoing efforts to characterize educational needs, knowledge gaps, perceptions, and readiness for AI integration across multiple learner populations. Intervention studies provided preliminary evidence supporting the effectiveness of AI-focused educational initiatives; however, relatively few investigations evaluated long-term competency acquisition or sustained educational outcomes.

In contrast, non-empirical studies primarily consisted of curriculum frameworks, expert recommendations, consensus statements, position papers, educational roadmaps, and theoretical guidance documents (39, 45, 47, 49, 54). While these publications contributed substantially to defining educational priorities and identifying essential competency domains, most lacked formal outcome assessment and therefore provided limited evidence regarding educational effectiveness. Nevertheless, these conceptual contributions have played a critical role in establishing the foundational frameworks that guide current curriculum development efforts.

**Figure 5:**
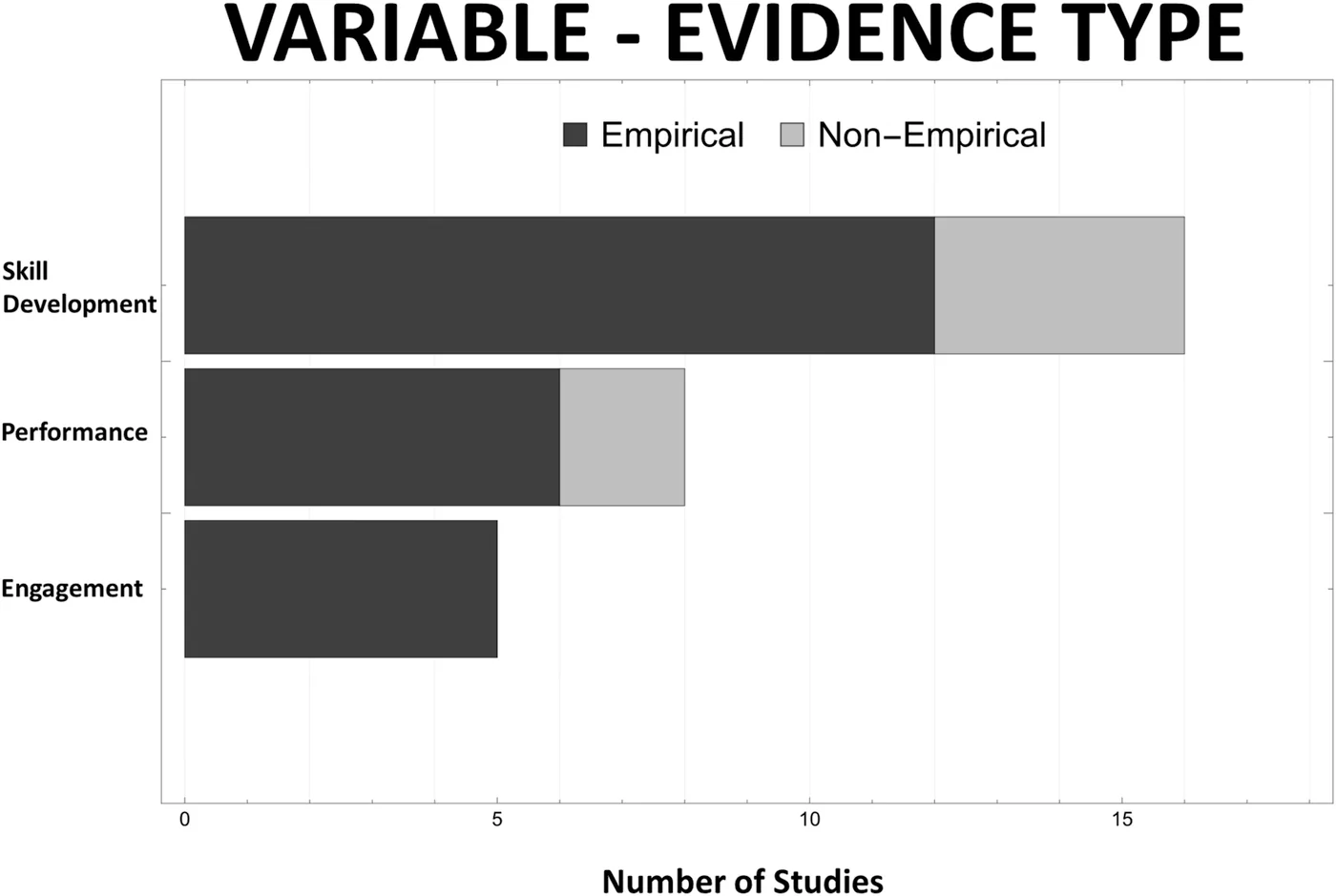
Relationship between educational priorities and evidence types in studies of artificial intelligence in radiology education.

Figure 5 illustrates the relationship between educational priorities and evidence types. Skill development emerged as the dominant focus across both empirical and non-empirical literature, demonstrating its central role in contemporary AI education research. Notably, empirical studies substantially outnumbered conceptual publications within this category, indicating increasing emphasis on evaluating educational effectiveness and measurable learner outcomes. Performance-related outcomes were investigated almost exclusively through empirical methodologies due to their reliance on objective measures such as diagnostic accuracy, examination performance, algorithm evaluation, and comparative testing (57, 60–63, 65). Similarly, engagement-related outcomes were entirely supported by empirical evidence and were predominantly derived from survey-based investigations assessing perceptions, attitudes, and readiness for AI adoption (55, 56, 58, 59, 64, 66).

### 3.2 Reported educational findings, readiness, and implementation barriers of AI in radiology education

**Figure 6:**
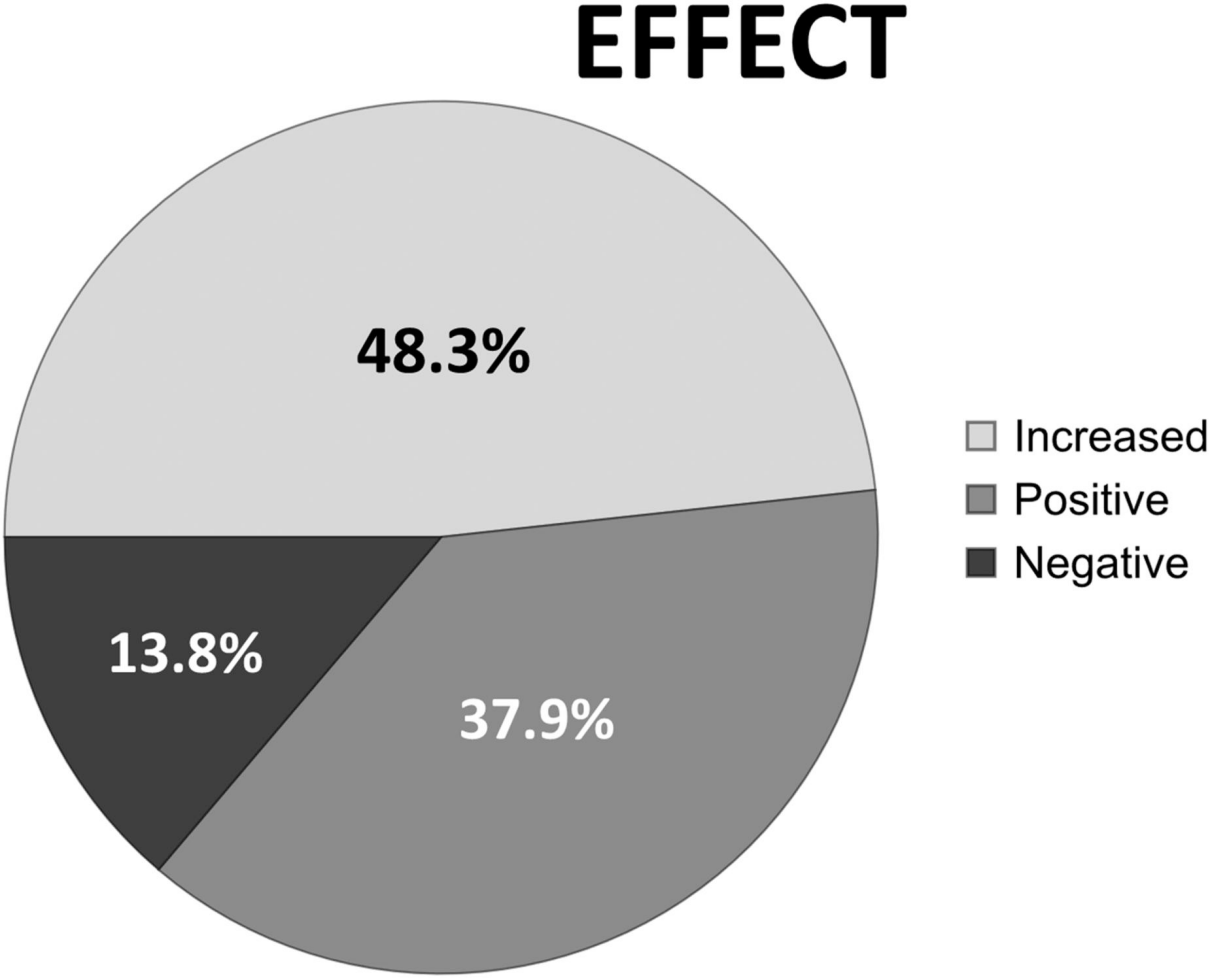
Distribution of reported educational effects among studies included in the scoping review of artificial intelligence in radiology education.

Table 4 summarizes the reported educational findings, learner readiness, implementation contexts, and barriers associated with AI in radiology education across the included studies. The findings reveal a predominantly favorable educational landscape, with most investigations reporting either positive outcomes or measurable improvements following exposure to AI-related educational interventions, training initiatives, or AI-assisted learning technologies. As illustrated in Figure 6, nearly half of the included studies reported increased educational outcomes (48.3%, *n* = 14), while an additional 37.9% (*n* = 11) described positive educational effects. In contrast, only 13.8% (*n* = 4) reported predominantly negative findings. Collectively, these results indicate that approximately 86% of the reviewed literature identified beneficial educational impacts associated with AI integration, suggesting strong potential for AI to enhance radiology education when appropriately implemented.

**Table 4:** Synthesis of included publications on artificial intelligence in radiology education, including technology type, reported effect, study or training context, and identified barriers.

Reference	AI/Machine Learning	Effect	Study/Training	Barriers
Almarzouki A.; et al. (55).	General AI concepts; AI in healthcare applications.	Negative	Medicine (MBBS)	Limited institutional AI resources; reliance on social media/informal sources.
Li R.; et al. (38).	AI-driven online training platform concept (technology not yet implemented; general AI).	Positive	Radiology/medical imaging education	Content creation burden; algorithm accuracy/interpretability; need cross-disciplinary staff and sustained funding; user trust in AI feedback.
Huang, D.; et al. (56).	AI in healthcare; ChatGPT awareness; AI-generated scientific text	Negative	Residency training (multi-specialty).	Low knowledge of clinical AI applications; ethical/data-security concerns; difficulty detecting AI-generated scientific writing.
Zanca, F.; et al. (39).	Artificial Intelligence including machine learning (ML), deep learning (DL), neural networks, radiomics, and AI-based image analysis systems	Positive	Medical physicists and trainees in medical physics	Lack of standardized AI training programs for medical physicists; limited existing educational resources tailored to medical physics needs
Genc, O.; et al. (57).	Large language models (ChatGPT-4o, Gemini Pro, Microsoft Copilot)	Positive	Clinician-facing decision/communication support (not a trainee curriculum)	Subjectivity in empathy scoring; low inter-rater agreement (ICC reported as poor/modest)
McFarlane A.; et al. (40).	AI in healthcare (general clinical/systems applications; decision support concepts)	Increased	Needs assessment for AI curriculum integration in residency/medical education	Perceived lack of physician agency in AI development; concerns about black-box opacity, over-reliance, and profit-driven implementation
Halat DH.; et al. (41).	AI in healthcare/dentistry (general); readiness framework (MAIRS domains: cognition, ability, vision, ethics)	Increased	Curriculum needs assessment for AI competencies in dental training	Moderate readiness with lower scores in cognition reportability; concerns about AI risks/disadvantages; limited prior AI training
Cheng, C.; et al. (42).	Artificial intelligence using deep learning and convolutional neural networks (CNNs) for fracture detection on radiographs	Increased	Medical students	Limited exposure to only one pathology and imaging modality
Pedersen M.; et al. (43).	AI in radiography, workflow efficiency, image processing, positioning, QA	Increased	Continuing professional development and curriculum integration needs	Limited educational opportunities; concern driven by lack of expertise/knowledge
Sharma V.; et al. (44).	Artificial intelligence applications in healthcare including machine learning–based diagnostic support systems and AI-driven clinical decision tools	Increased	Medical students	Limited faculty expertise, lack of educational resources, and concerns about the impact of AI on clinical skills
Laborie L.; et al. (45).	AI/ML in pediatric imaging	Increased	Training strategy and collaborative network-building (multicenter datasets, multi-reader studies)	Time, funding, and limited guidance/mentorship for education; paucity of pediatric-tailored tools
Hu R.; et al. (46).	Intro AI/ML/DL concepts; model validation; common ML models; CNN/GAN familiarity; Google Colab demos	Increased	Diagnostic radiology residents	Limited protected time and heterogeneous baseline technical background; potential selection bias due to voluntary attendance
Tejani A.; et al. (47).	ML/DL fundamentals; NLP, computer vision; governance/ethics; AI deployment/monitoring; GAN-based case synthesis (conceptual)	Positive	Radiology trainees and practicing radiologists	Access to mentors; limited time; limited trainee interest; heterogeneous trainee experience; rapid technology changes
Staziaki P.; et al. (48).	Artificial intelligence applied to radiology; critical appraisal of the scientific literature, peer review, and scholarly communication	Positive	Medical students, residents, fellows, and research trainees	Need for training in scientific peer review and critical evaluation of AI research
Ngo B.; et al. (49).	Artificial intelligence, general; ML/DL concepts	Positive	Medical students	Crowded curriculum; limited faculty expertise; lack of consensus on what/how to teach; uncertain near-term clinical usefulness
Lindqwister, A.; et al. (50).	Machine learning. foundational algorithms; basic computing concepts	Increased	Radiology residents	Time constraints; engagement/attrition; lack of external requirements (e.g., board/ACGME) may reduce prioritization
Lindqwister, A.: et al. (51).	Machine learning foundational algorithms	Increased	Training	Time constraints; declining journal club preparation; scheduling conflicts
Rainey, C.; et al. (52).	General AI/ML and deep learning concepts/terminology in radiography practice	Negative	Study	Perceived insufficient training/skills and low confidence for clinical AI implementation
Ooi, S.; et al. (58).	Artificial intelligence; machine learning: radiology applications, informatics.	Increased	Needs assessment/curriculum planning	Low self-rated AI/ML familiarity; perceived inadequate program implementation of AI/ML teaching.
Sit, C.; et al. (59).	Artificial intelligence, including deep learning/CNN-based radiology tools.	Negative	Attitudes/education needs assessment	Minimal/optional AI education; low confidence in future critical use of AI tools; career deterrence in radiology.
Bin Dahmash A.; et al. (53).	Artificial intelligence in medical imaging; general AI concepts rather than evaluation of a specific algorithm	Positive	Radiology residents/fellows	Limited formal AI education and misconceptions regarding the role of AI in radiology
Recht, M.; et al. (54).	Machine/deep learning for imaging CADe/CADx/CADt; workflow integration	Positive	Practicing radiologists and radiology trainees	Bias/FAIRness issues; privacy and re-identification risk; incentives/cost and competition limiting data sharing; regulatory hurdles; insufficient AI literacy
Mithun S.; et al. (60).	Deep learning NLP models (Bi-LSTM, Bi-LSTM with dropout, and BERT) for automated classification of lung cancer radiology reports	Increased	Radiology/Lung Cancer Imaging	Variability in report language and terminology; dependence on annotated datasets; challenges in external generalizability.
Huang Y.; et al. (61).	ChatGPT-3.5 and ChatGPT-4 for radiation oncology education and clinical decision support	Increased	Radiation Oncology	Risk of hallucinations; lower performance in some domains (e.g., brachytherapy, clinical trials); need for expert verification.
Chen Y.-S.; et al. (62).	Deep learning AI algorithm (Faster-RCNN with Inception-ResNet-v2) for detecting impacted animal bones on lateral neck radiographs	Increased	Radiology, Emergency Medicine, ENT	Limited labeled datasets; privacy/data protection concerns; AI interpretability issues; lack of clinical AI guidelines.
Park S.; et al. (63).	Deep learning model using transfer learning and semi-supervised learning (DISTL, Vision Transformer) for pneumoperitoneum detection	Increased	Radiology	Limited labeled training data; challenges in generalizability across institutions; difficulty detecting subtle findings.
Hamd Z.Y.; et al. (64).	Artificial Intelligence (AI), Machine Learning (ML), Deep Learning (DL), neural-network–based dental applications, image analysis, speech recognition, data mining.	Positive	Dentistry (students, dentists, academic staff)	Lack of formal AI education and training programs; low organizational readiness; uncertainty about AI implementation strategies; insufficient institutional support and infrastructure for AI adoption.
Mago J.; et al. (65).	ChatGPT-3 (Large Language Model, Natural Language Processing) applied to oral and maxillofacial radiology.	Positive	Oral and Maxillofacial Radiology education/training	Limited detail and specificity of responses; incorrect interpretation of abbreviations; risk of misinformation (“infodemics”); ethical, copyright, legal, and regulatory concerns; unsuitable as a primary training resource.
Jackson P.; et al. (66).	General AI applications in healthcare, expert systems, machine learning, neural networks, image analysis, virtual assistants, AI-assisted diagnosis.	Positive	Undergraduate Medical Education (MBBS)	Very limited prior AI training (91.4% had none); low self-reported AI competence; concerns about job displacement, reduced humanistic care, patient-physician relationship damage, trust issues, confidentiality breaches, and ethical challenges.

The predominance of increased and positive outcomes reflects growing evidence that AI-related educational initiatives can improve learner knowledge, confidence, awareness, and preparedness for AI-enabled clinical practice (38–51, 53, 54, 57, 58, 60–65). Across undergraduate medical education, radiology residency programs, radiography training, medical physics education, and continuing professional development settings, participants consistently demonstrated greater familiarity with AI concepts following educational exposure. Structured educational interventions focusing on machine learning, deep learning, model validation, clinical implementation, and AI governance were associated with enhanced understanding of AI principles and increased confidence in engaging with AI technologies (46, 50, 51). Similarly, AI-assisted learning tools designed to support image interpretation and diagnostic reasoning demonstrated educational benefits by facilitating knowledge acquisition and improving learner performance (42).

The positive educational impact of AI was observed not only in formal curricula but also in conceptual frameworks and strategic educational initiatives. Curriculum development studies emphasized the importance of integrating AI competencies into radiology training pathways through progressive and competency-based educational models (39, 45, 47, 49). These studies highlighted the need to prepare future radiologists for emerging clinical environments in which AI is increasingly embedded within imaging workflows, decision-support systems, and diagnostic processes. Furthermore, initiatives promoting critical appraisal skills, scientific literacy, and AI research evaluation suggested that educational programs should extend beyond technical instruction to include the interpretation, validation, and responsible use of AI technologies (48).

A prominent theme across the literature was the assessment of learner readiness for AI adoption. Despite generally positive perceptions toward AI, many studies identified substantial gaps in knowledge, confidence, and formal training (40, 41, 43, 44, 52, 58, 66). Participants frequently recognized AI as an inevitable component of future healthcare delivery and expressed strong interest in acquiring AI-related competencies. However, self-reported preparedness remained relatively low across multiple learner groups, including medical students, radiology residents, radiographers, and practicing healthcare professionals (43, 52, 58, 66). These findings suggest that enthusiasm for AI adoption often exceeds current educational preparedness, highlighting a critical need for structured learning opportunities and competency-based training programs. Several studies specifically investigated educational needs and readiness frameworks. The findings consistently demonstrated moderate readiness levels accompanied by limited technical knowledge and insufficient understanding of AI applications in clinical practice (41, 43, 58). Learners commonly reported uncertainty regarding algorithm interpretation, model validation, ethical considerations, and the practical integration of AI into diagnostic workflows. While the literature indicates strong willingness to engage with AI, readiness for implementation remains constrained by educational deficiencies rather than resistance to technological innovation.

The emergence of generative AI and large language models has introduced additional opportunities and challenges within radiology education. Studies evaluating platforms such as ChatGPT, Gemini, and Copilot reported encouraging educational performance, particularly in areas related to information retrieval, clinical communication, examination preparation, and self-directed learning (57, 61, 65). These technologies demonstrated the potential to serve as accessible educational resources capable of supporting learners across multiple stages of training. However, researchers consistently identified concerns regarding hallucinations, factual inaccuracies, incomplete clinical reasoning, and variability in response quality (61, 65). As a result, the literature strongly supports the use of generative AI as a supplementary educational tool rather than a replacement for expert instruction, clinical supervision, or evidence-based educational resources.

Although positive findings predominated, a minority of studies reported negative outcomes or identified significant deficiencies that may impede successful implementation (52, 55, 56, 59). These studies highlighted low levels of AI literacy, inadequate understanding of clinical AI applications, insufficient confidence in evaluating AI tools, and concerns regarding the future impact of AI on professional roles. In particular, some learners perceived AI as a potential threat to radiology careers, reflecting persistent misconceptions regarding the relationship between AI systems and healthcare professionals (59). Such concerns underscore the importance of educational programs that not only teach technical concepts but also address professional identity, workforce transformation, and human–AI collaboration.

The most consistently reported barrier across the literature was the lack of formal AI education and training opportunities. Educational deficiencies were documented across nearly all learner groups, including medical students, residents, radiographers, faculty members, and practicing clinicians (43, 52, 53, 55, 59, 64, 66). Many participants reported obtaining AI-related knowledge through informal sources such as social media, online videos, independent reading, or peer discussions rather than structured educational programs (55). This reliance on unregulated information sources may contribute to inconsistent knowledge acquisition and variable levels of AI literacy.

Faculty expertise and educational capacity emerged as additional barriers to implementation (39, 44, 49, 52, 58). Many institutions lacked instructors with sufficient AI knowledge to develop and deliver comprehensive educational programs. The rapid evolution of AI technologies further complicates faculty development efforts, requiring ongoing professional training to ensure curricular relevance and educational quality. Consequently, limited faculty expertise remains a significant obstacle to the widespread integration of AI education within radiology training programs.

Curricular constraints also represented a major implementation challenge. Several studies reported difficulties incorporating AI content into already crowded medical and radiology curricula (49). Time limitations, competing educational priorities, and demanding clinical responsibilities restricted opportunities for dedicated AI instruction (46, 47, 50, 51). Also, maintaining learner engagement throughout longitudinal educational interventions proved challenging in some residency-based programs (50, 51). These findings suggest that successful implementation may require flexible and longitudinal educational models that integrate AI concepts across existing curricula rather than introducing isolated courses.

Technical and organizational barriers were likewise frequently reported. Challenges included insufficient funding, limited institutional support, inadequate computational infrastructure, restricted access to high-quality datasets, and difficulties establishing interdisciplinary collaborations (38, 45, 64). Several studies emphasized the importance of partnerships between radiologists, educators, computer scientists, engineers, and data scientists to support curriculum development and AI implementation (38, 45). Additionally, heterogeneity in learners' technical backgrounds created difficulties in designing educational experiences that effectively accommodate varying levels of prior knowledge and computational expertise (46, 47). Ethical, legal, and regulatory considerations constituted another major barrier category. Concerns regarding algorithmic bias, transparency, explain ability, privacy protection, data governance, accountability, and regulatory oversight were frequently reported (54, 56, 62, 64–66). The increasing use of generative AI further introduced concerns regarding misinformation, academic integrity, copyright issues, intellectual property rights, and the reliability of AI-generated educational content (61, 65). These findings highlight the necessity of incorporating ethical reasoning, governance principles, and critical appraisal skills into AI curricula alongside technical competencies.

Some studies identified technological barriers related to AI development and deployment, including limited labeled datasets, challenges in external validation, variability in imaging data, and concerns regarding model generalizability across institutions and patient populations (60, 62, 63). Although primarily associated with AI implementation rather than education itself, these limitations have important educational implications because radiology professionals must understand such constraints to critically evaluate AI tools and ensure safe clinical adoption.

### 3.3 Educational focus and training approaches in artificial intelligence for radiology education

**Figure 7:**
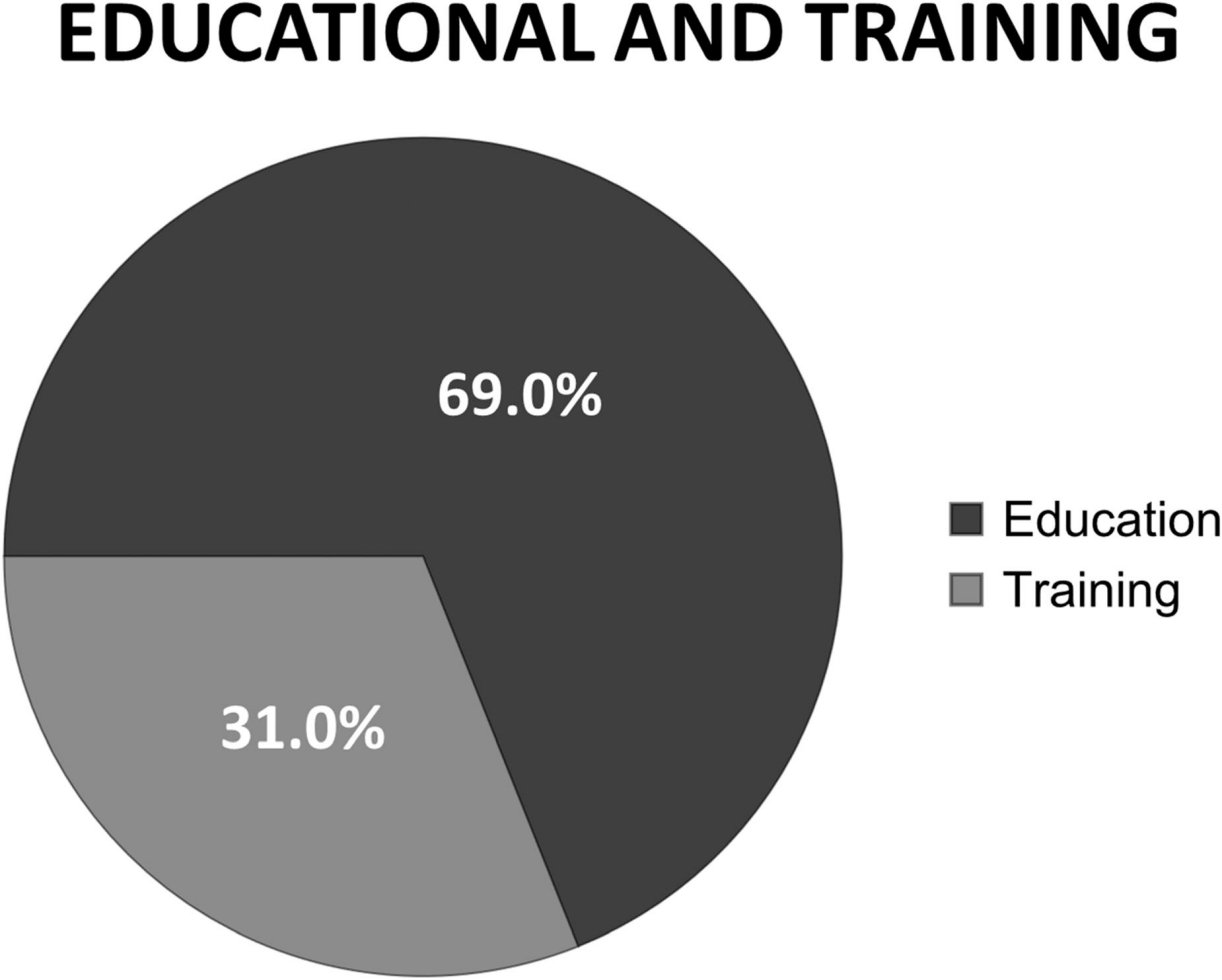
Distribution of educational focus among studies included in the scoping review of artificial intelligence in radiology education.

Table 5 shows summarizes the educational stages, instructional focus, and methodological limitations of the studies included in this review. The analysis demonstrates that AI-related initiatives in radiology education have been implemented across multiple educational levels, ranging from undergraduate medical and dental education to residency training, graduate medical education, professional development programs, and clinical practice settings. As illustrated in Figure 7, educationally oriented studies constituted the majority of the literature, representing 69.0% of included publications (*n* = 20), whereas studies primarily focused on training interventions accounted for 31.0% (*n* = 9). This distribution suggests that current research has largely concentrated on establishing foundational AI literacy, awareness, and conceptual understanding, while comparatively fewer investigations have evaluated structured competency-based training programs designed to develop practical AI skills.

**Table 5:** Educational stage, educational or training focus, and reported limitations across the included publications on artificial intelligence in radiology education.

Reference	Stage	Training/Education	Limitations
Almarzouki A.; et al. (55).	Undergraduate dental education	Education	Cross-sectional; self-reported knowledge; convenience sampling; limited generalizability to Saudi Arabia.
Li R.; et al. (38).	Undergraduate dental education	Training	Self-selected single-center sample; questionnaire option constraints limiting nuanced viewpoints.
Huang, D.; et al. (56).	Postgraduate medical residents	Education	Potential underestimation of anxiety; discrimination task used only conclusion sections; small subsamples by specialty limit subgroup analyses.
Zanca, F.; et al. (39).	Professional and postgraduate training	Training	Conceptual proposal without evaluation of educational outcomes; curriculum may require periodic updates due to rapid evolution of AI technologies
Genc, O.; et al. (57).	Clinical practice	Education	Single comparator physician; limited number of evaluators and questions; outcomes based on expert ratings rather than patient comprehension or clinical endpoints.
McFarlane A.; et al. (40).	Graduate medical education	Education	Single-site sample; no surgical specialties/dermatology; perceptions reflect 2018–2019 context; voluntary participation may bias toward interested residents.
Halat DH.; et al. (41).	Undergraduate dental education	Education	Single-institution sample; self-reported measures; response rate below estimated representative target; cross-sectional design prevents causal inference.
Cheng, C.; et al. (42).	Undergraduate medical education	Training	Small sample size and single-institution study
Pedersen M.; et al. (43).	Radiographers and radiography students (Nordic countries)	Education	English-only survey; skewed geography (≈2/3 Denmark); incomplete responses; potential selection/sequencing bias; online distribution limits representativeness
Sharma V.; et al. (44).	Undergraduate medical education	Education	Online survey methodology with possible selection bias; participation uneven across regions; inclusion limited to students only
Laborie L.; et al. (45).	Pediatric radiologists and imaging departments	Education	Descriptive/strategic (not outcomes-based); goals may evolve with regulation/technology
Hu R.; et al. (46).	Postgraduate	Education	Single institution/small sample; no objective knowledge test; knowledge retention not assessed; possible selection bias
Tejani A.; et al. (47).	Undergraduate–Postgraduate–CME	Education	Non-empirical (no outcome evaluation); recommendations may require frequent updating as technologies/regulation evolve
Staziaki P.; et al. (48).	Undergraduate and graduate medical education	Training	Descriptive report: participant self-selection and competitiveness may limit generalizability; limited long-term outcomes reporting
Ngo B.; et al. (49).	Undergraduate	Education	Non-empirical (opinion-based); no outcomes evaluation; generalizability depends on local curricula
Lindqwister, A.; et al. (50).	Graduate medical education	Education	Small sample; attrition; subjective immediate pre–post measures; no matched control group; limited evidence on long-term retention
Lindqwister, A.: et al. (51).	Residency	Training	Heterogeneous attendance due to rotations/scheduling and resident burnout; no formal objective testing
Rainey, C.; et al. (52).	Undergraduate dental education	Education	Convenience/snowball sampling → limited generalizability; potential selection bias toward AI-interested respondents
Ooi, S.; et al. (58).	Residency	Education	Self-reported attitudes; cross-sectional design; potential response/selection bias.
Sit, C.; et al. (59).	Undergraduate medical school	Education	Not all UK schools represented; self-selection and response bias; cross-sectional design.
Bin Dahmash A.; et al. (53).	Undergraduate medical education	Education	Cross-sectional design, self-reported responses, and potential selection bias
Recht, M.; et al. (54).	Clinical implementation	Education	No empirical outcomes: recommendations may be context-dependent; implementation feasibility not tested
Mithun S.; et al. (60).	Professional	Training	Single-center training dataset; variability in radiology report language; class imbalance; reduced performance on external validation; dependence on annotated datasets.
Huang Y.; et al. (61).	Residency	Education	Exclusion of image-based questions; risk of hallucinations; weaker performance in brachytherapy and clinical trials; limited to evaluated ChatGPT versions.
Chen Y.-S.; et al. (62).	Professional	Training	Retrospective single-center study; limited labeled datasets; privacy concerns; AI interpretability issues; lack of clinical AI guidelines.
Park S.; et al. (63).	Residency	Training	Retrospective design; limited labeled training data; generalizability concerns across institutions; false negatives with small amounts of free air.
Hamd Z.Y.; et al. (64).	Undergraduate dental education	Education	Cross-sectional design; self-reported knowledge and perceptions; conducted only in the UAE; limited generalizability; organizational readiness not objectively measured.
Mago J.; et al. (65).	Oral and Maxillofacial Radiology training	Training	Single evaluator; only 80 questions assessed; limited to ChatGPT-3; no comparison with human learners or other AI systems.
Jackson P.; et al. (66).	Undergraduate medical education	Education	Single-institution study; voluntary participation; self-reported perceptions and knowledge; cross-sectional design; limited generalizability.

The predominance of educational approaches reflects the developmental stage of AI integration within radiology curricula. Most studies focused on introducing learners to fundamental AI concepts, including machine learning, deep learning, neural networks, radiomics, clinical applications of AI, ethical considerations, governance frameworks, and the potential impact of AI on healthcare delivery (40–47, 49, 50, 52–59, 61, 64, 66). These initiatives were primarily designed to improve awareness, increase AI literacy, and prepare learners to critically engage with emerging technologies. Across undergraduate, postgraduate, and professional contexts, researchers consistently identified a need for greater foundational knowledge of AI principles before advanced implementation-focused competencies could be effectively developed (40, 43, 44, 55, 58, 59, 66).

Undergraduate medical and dental education represented one of the most frequently investigated educational stages (38, 41, 42, 44, 49, 52, 53, 55, 59, 64, 66). Within this population, studies commonly explored learner perceptions, knowledge gaps, attitudes toward AI integration, and curriculum needs. Findings consistently demonstrated strong interest in AI education accompanied by limited prior exposure to AI-related content (41, 44, 55, 59, 64, 66). Many students recognized the growing importance of AI within healthcare and expressed support for its inclusion within formal curricula. However, self-reported competence and confidence in understanding AI technologies remained relatively low, emphasizing the need for structured educational frameworks capable of introducing foundational concepts early in medical training (44, 49, 59, 66). Postgraduate and residency-based education constituted another major area of investigation (46, 51, 56, 58, 61, 63). These studies primarily focused on preparing future radiologists and medical specialists to navigate AI-enabled clinical environments. Educational interventions implemented at the residency level frequently included lectures, workshops, journal clubs, case discussions, and technology demonstrations designed to enhance understanding of machine learning algorithms, model validation, clinical applications, and AI-assisted decision-making (46, 51). The literature suggests that residency programs represent a critical stage for AI education because learners are simultaneously developing clinical expertise and acquiring professional competencies necessary for independent practice. Consequently, several studies advocated for the integration of AI-related competencies into residency curricula through longitudinal educational models that align technological knowledge with clinical training (46, 51, 58).

Graduate medical education and professional development initiatives also emerged as important educational contexts (39, 40, 48, 60, 62). These studies frequently emphasized advanced competency development, critical appraisal skills, research literacy, and the practical implementation of AI technologies in clinical workflows. Professional-level initiatives often focused on preparing practicing radiologists, radiographers, medical physicists, and imaging specialists to critically evaluate AI systems, understand algorithmic limitations, and participate in technology adoption processes (39, 48). Such findings highlight the necessity of lifelong learning approaches to AI education, given the rapid pace of technological innovation and the continuous evolution of AI applications in radiology.

While educational initiatives dominated the literature, a substantial proportion of studies focused specifically on training-oriented approaches (38, 39, 42, 48, 51, 60, 62, 63, 65). These investigations typically emphasized skill acquisition through hands-on learning experiences, competency development, AI-assisted image interpretation, model evaluation, and practical exposure to AI technologies. Unlike educational studies that focused primarily on conceptual understanding, training-focused studies sought to develop operational competencies and applied knowledge. For example, AI-assisted diagnostic systems, machine learning workshops, and algorithm evaluation exercises were designed to facilitate experiential learning and improve learner performance in clinically relevant contexts (42, 60, 62, 63). These findings suggest that while awareness and literacy remain important objectives, future educational efforts may increasingly shift toward competency-based training models that provide learners with practical experience in AI utilization and evaluation.

The distribution illustrated in Figure 7 further supports this interpretation. The larger proportion of education-focused studies indicates that AI curricula remain largely concentrated on foundational knowledge acquisition rather than advanced technical skill development. This pattern is consistent with broader trends in medical education, where curricular reform often begins with awareness-building and conceptual instruction before progressing toward competency assessment and practical application. The current evidence suggests that radiology education is undergoing a similar transition, with increasing emphasis on moving from theoretical understanding toward implementation-oriented training.

Despite the diversity of educational stages and instructional approaches represented in the literature, methodological limitations were consistently reported across studies. One of the most common limitations involved the widespread reliance on cross-sectional study designs and self-reported measures (41, 52, 53, 55, 59, 64, 66). While such methodologies are valuable for assessing perceptions, attitudes, and educational needs, they provide limited evidence regarding actual competency acquisition, behavioral change, or long-term educational effectiveness. Consequently, many studies were unable to determine whether reported improvements in confidence or perceived knowledge translated into meaningful clinical competencies. Small sample sizes and single-institution recruitment strategies were also frequently identified as limitations (38, 40, 42, 46, 50, 66). These factors may reduce the generalizability of findings and limit the applicability of results across different educational systems, institutions, and geographic regions. Similarly, convenience sampling and voluntary participation may have introduced selection bias by disproportionately attracting participants with preexisting interest in AI-related topics (40, 52, 55, 59). Such biases may contribute to overly favorable perceptions of AI education and potentially underestimate barriers encountered among broader learner populations.

Several studies additionally reported limitations related to outcome assessment and program evaluation (39, 45, 47, 49, 54). Non-empirical publications frequently proposed educational frameworks, curricular recommendations, or strategic roadmaps without formally evaluating educational outcomes. Although these contributions provide valuable guidance for curriculum development, they offer limited evidence regarding the effectiveness of proposed educational approaches. Similarly, intervention studies often lacked objective testing, control groups, long-term follow-up, or assessments of knowledge retention, restricting conclusions regarding the sustainability of educational gains (46, 50, 51).

## 4 Discussion

### 4.1 Evolution from awareness to competency

**SCHEME 1:**
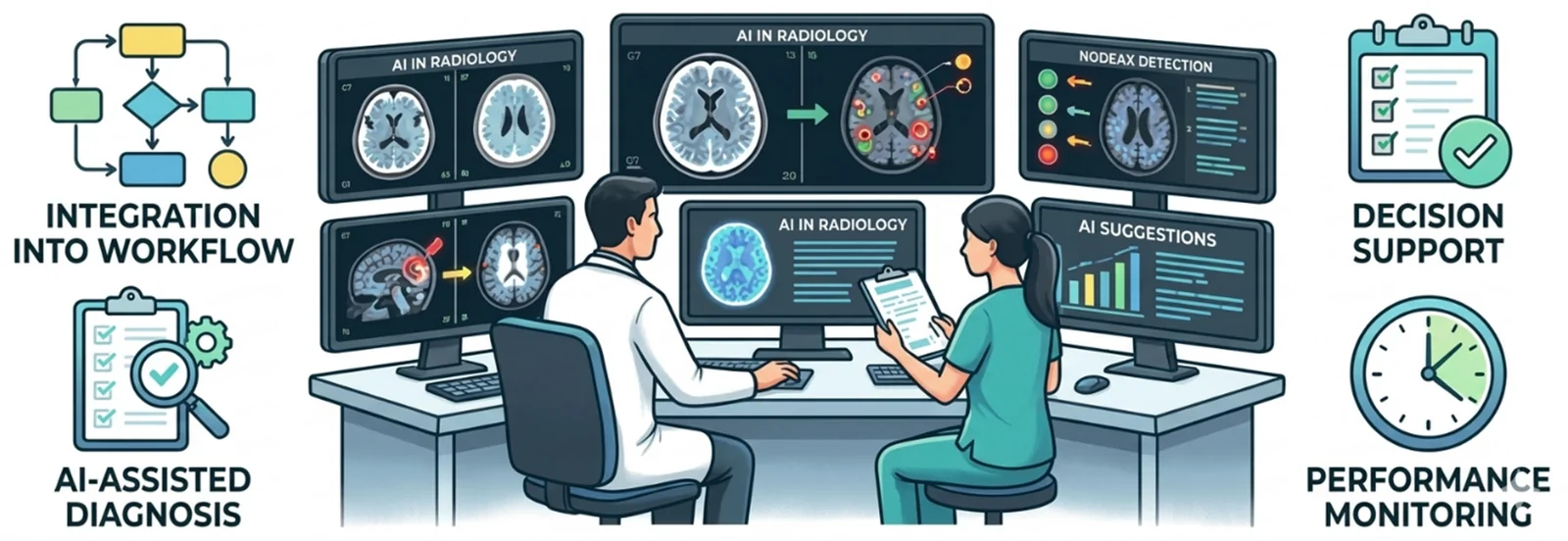
Artificial intelligence applications and competencies relevant to radiology education and practice.

Early discussions surrounding AI in healthcare education were largely centered on understanding learner attitudes, concerns regarding professional displacement, and the anticipated impact of AI on future clinical practice (Scheme 1). However, the contemporary literature demonstrates a clear shift toward the development of educational frameworks, training programs, and competency-based curricula designed to prepare radiology professionals for AI-enabled healthcare environments. This evolution reflects the growing recognition that AI is no longer a future consideration but an increasingly integrated component of radiological practice requiring formal educational preparation.

The progression from awareness to competency is evident in the distribution of educational priorities identified in the included studies. More than half of the reviewed publications focused on skill development, whereas engagement-related outcomes represented the smallest proportion of the literature. This pattern suggests that the field has moved beyond merely assessing learner perceptions toward evaluating how AI-related competencies can be developed and integrated into radiology training programs. Early investigations primarily explored awareness, attitudes, and readiness for AI adoption among medical students, residents, and imaging professionals (55, 56, 58, 59, 64, 66). These studies consistently reported high levels of interest in AI coupled with limited formal training, low self-reported competence, and uncertainty regarding AI implementation in clinical practice. Such findings highlighted a substantial educational gap and provided the initial rationale for curriculum development efforts.

The widespread lack of AI preparedness observed across learner populations appears to have catalyzed the development of more structured educational initiatives. Multiple studies emphasized the need for formal AI instruction covering machine learning, deep learning, algorithm interpretation, clinical implementation, and ethical governance (39, 45, 47, 49, 54). Importantly, these publications moved beyond identifying educational deficiencies and instead proposed frameworks for competency acquisition and curriculum integration. The emergence of structured educational roadmaps and competency frameworks suggests increasing consensus regarding the necessity of AI literacy as a core professional competency for radiologists and imaging specialists.

A notable finding of this review is the increasing diversity of educational approaches employed to address AI competency development. Several studies described educational interventions ranging from short workshops and lecture series to longitudinal residency curricula and professional development programs (46, 50, 51). These interventions typically introduced foundational concepts such as machine learning algorithms, neural networks, model validation, and AI-assisted clinical decision-making while also addressing practical considerations related to implementation and governance. The positive educational outcomes reported across these initiatives suggest that structured learning experiences can improve learner confidence, knowledge, and readiness for AI integration. However, the predominance of self-reported outcomes and the limited use of objective competency assessments indicate that the field remains in the early stages of evaluating educational effectiveness.

Another important development identified in this review is the expansion of AI education beyond traditional radiology trainees. Educational initiatives were implemented across undergraduate medical education, residency training, graduate medical education, radiography programs, medical physics training, and continuing professional development settings (38–66). This broad distribution reflects recognition that AI competency is not confined to a single professional group but represents a multidisciplinary requirement across the imaging workforce. The inclusion of radiographers, medical physicists, educators, and practicing radiologists within AI educational initiatives further supports the concept of lifelong learning as a critical component of AI integration in healthcare.

The emergence of generative AI and large language models has further accelerated the evolution of AI education. Studies evaluating systems such as ChatGPT, Gemini, and Copilot demonstrated promising capabilities in educational support, clinical communication, and knowledge assessment (57, 61, 65). These technologies have expanded the scope of AI education by introducing new competencies related to prompt engineering, critical evaluation of AI-generated content, verification of outputs, and responsible use of generative systems. At the same time, concerns regarding hallucinations, misinformation, bias, and reliability have reinforced the importance of critical appraisal skills and human oversight (61, 65), AI competency is increasingly understood as extending beyond technical knowledge to include ethical reasoning, governance awareness, and the ability to critically evaluate AI outputs within clinical contexts.

Despite these advances, the transition from awareness to competency remains incomplete. Many studies continued to identify substantial barriers related to limited faculty expertise, inadequate educational resources, crowded curricula, insufficient institutional support, and lack of standardized competency frameworks (38, 44, 47, 49, 52, 58, 64). Most educational interventions relied on short-term evaluations and self-reported measures, limiting understanding of long-term knowledge retention and competency acquisition. The absence of universally accepted educational standards also contributes to considerable heterogeneity in curriculum content, learning objectives, and assessment strategies. Taken together, the evidence indicates that AI education in radiology has progressed substantially from an initial phase characterized by awareness-building and exploratory discussions toward a more mature stage focused on competency development and workforce preparation. The literature increasingly supports the integration of structured, longitudinal, and competency-based AI education across all levels of radiology training.

### 4.2 Educational effectiveness and learner preparedness

Most educational interventions reported improvements in learner knowledge, confidence, awareness, and perceived readiness to engage with AI-enabled healthcare environments (38, 39, 42, 46, 50, 51). Positive outcomes were observed across medical students, radiology residents, radiographers, medical physicists, and practicing professionals, suggesting that AI education is relevant throughout the imaging workforce. But, much of the evidence was based on short-term interventions and self-reported outcomes, limiting conclusions regarding objective competency acquisition and sustained clinical performance.

**SCHEME 2:**
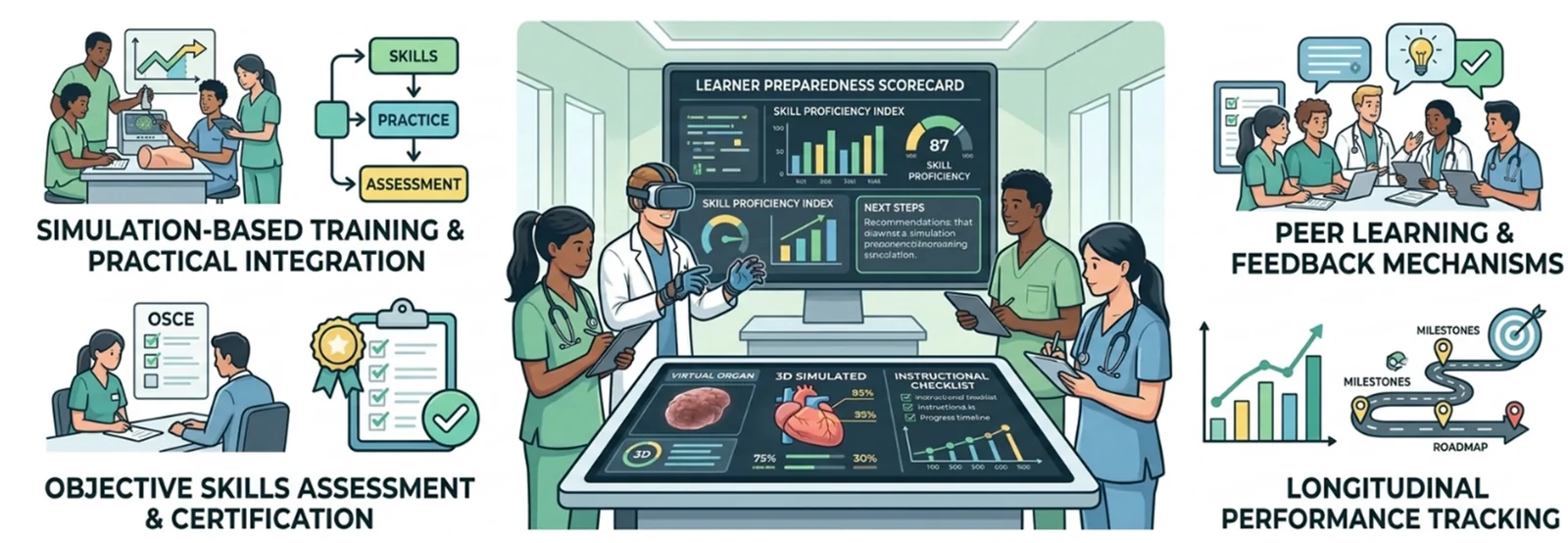
Proposed model for developing and assessing learner preparedness in artificial intelligence for radiology education.

Despite strong interest in AI, learners frequently reported limited formal exposure, low confidence in evaluating AI systems, and insufficient understanding of their clinical applications (43, 44, 52, 53, 55, 56, 58, 59, 64, 66). This gap between enthusiasm and preparedness indicates that introductory awareness alone is insufficient. Learners require opportunities to interpret algorithmic outputs, identify limitations and bias, and apply AI knowledge in clinically relevant situations (see Scheme 2). The increasing use of generative AI also introduces competencies related to output verification, hallucination recognition, ethical reasoning, and responsible use (57, 61, 65).

### 4.3 Competency-based and applied training approaches

The reviewed literature indicates a transition from awareness-oriented instruction toward structured and competency-based training. Interventions included residency curricula, workshops, journal clubs, AI-assisted learning platforms, case-based activities, and algorithm-evaluation exercises (38, 39, 42, 46, 48, 50, 51, 60, 62, 63, 65). These approaches sought to connect foundational concepts—including machine learning, model validation, clinical implementation, and ethics—with practical interaction with AI systems.

**SCHEME 3:**
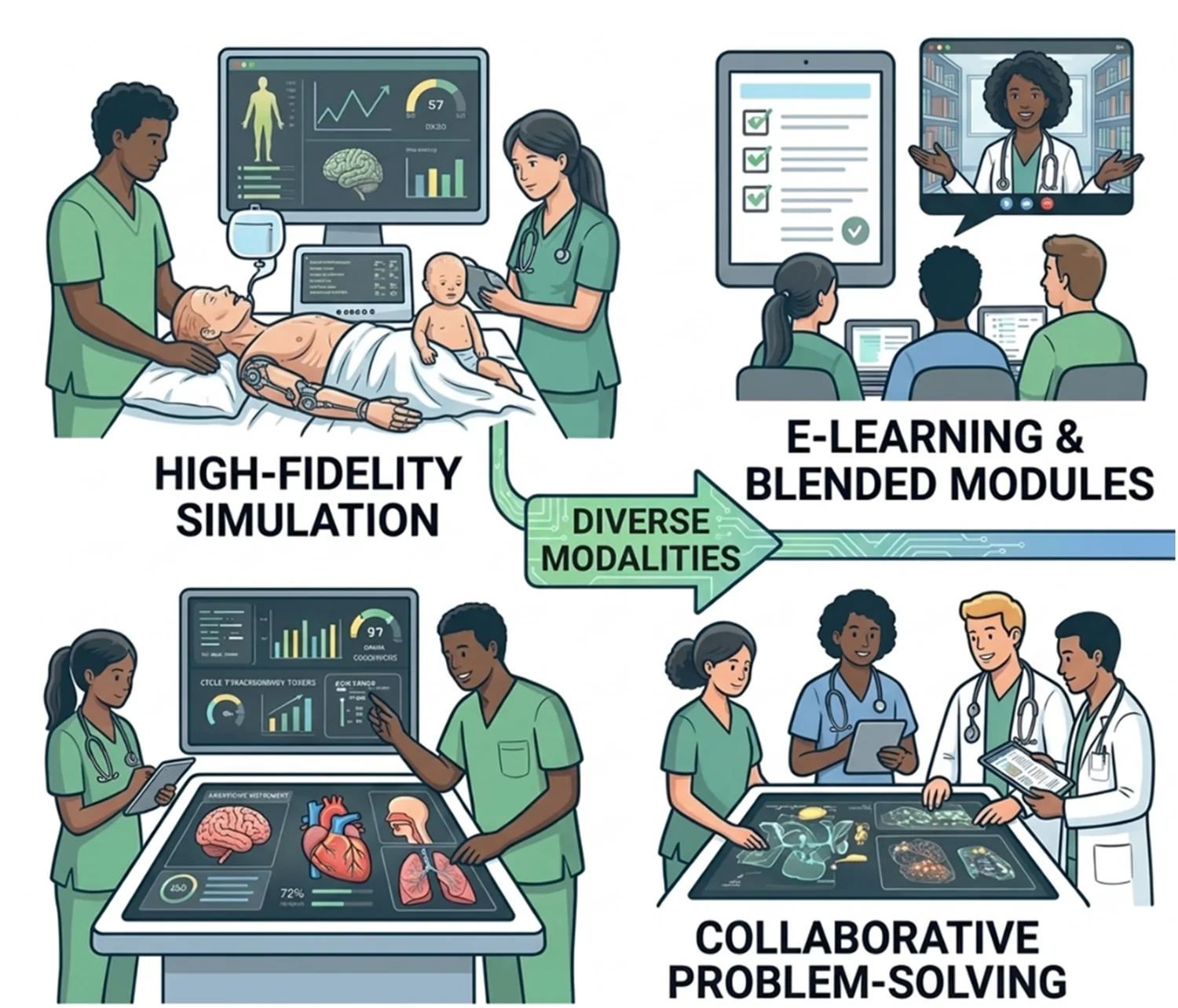
Framework of competency-based training approaches for artificial intelligence in radiology education.

Applied learning is particularly important because effective AI use requires radiology professionals to determine when algorithmic outputs should be accepted, questioned, verified, or rejected. Nevertheless, training programs remain heterogeneous, and objective competency assessments and longitudinal evaluations are uncommon. Future curricula should therefore define measurable learning outcomes and incorporate practical exercises, simulation, critical appraisal, objective assessment, and progressive competency development across undergraduate, postgraduate, and continuing professional education (see Scheme 3).

### 4.4 Institutional barriers and the role of structured pedagogy

Implementation is constrained by limited faculty expertise, crowded curricula, inadequate technological infrastructure, restricted access to datasets and AI tools, and variable institutional readiness (38, 39, 44, 45, 47, 49, 52, 58, 64). Faculty development is particularly important because educators must translate computational concepts into clinically meaningful instruction. Without appropriate training and support, AI education may remain fragmented, superficial, or dependent on informal self-directed learning.

**SCHEME 4:**
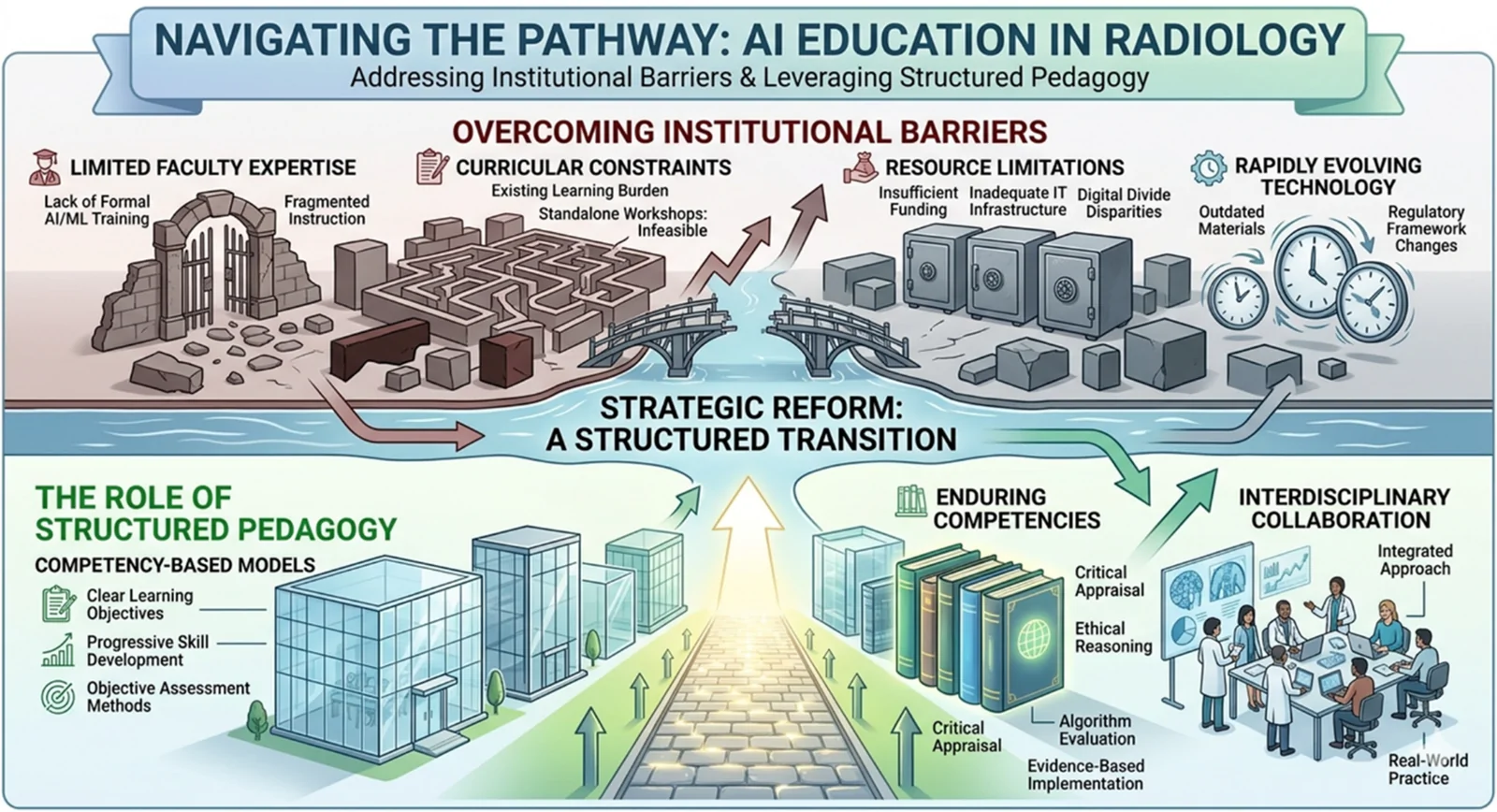
Conceptual framework for overcoming institutional barriers and advancing structured artificial intelligence education in radiology.

AI content should be integrated longitudinally into existing radiology curricula rather than added solely through isolated workshops or electives (Scheme 4). Because specific technologies evolve rapidly, educational programs should prioritize enduring competencies, including critical appraisal, model evaluation, bias recognition, ethical reasoning, governance, and evidence-based implementation. Sustainable adoption will require clearly defined learning objectives, interdisciplinary collaboration, adequate institutional resources, and assessment strategies aligned with competency-based education.

### 4.5 Comparison with prior reviews and related literature

While earlier reviews have generally focused on specific dimensions of AI education—such as competency frameworks, curriculum design, stakeholder perceptions, educational technologies, or technical developments in medical imaging—our review provides a comprehensive evaluation of educational priorities, learner preparedness, training approaches, educational effectiveness, and implementation barriers across the continuum of radiology education (see Table 6). This integrated perspective enables a more nuanced understanding of how AI is being incorporated into radiology training and where future educational efforts should be directed.

**Table 6:** Comparative summary of studies evaluating artificial intelligence in radiology and medical education.

Reference	Study Type	Educational Context	Main Variables/Outcomes	Key Findings
Volin et al. (67)	Narrative review/perspective	Radiology residency education	AI literacy, curriculum implementation, clinical AI exposure, ethics	Focuses on integrating AI into residency through structured curricula, modular learning, self-directed resources, and ethical training; highlights challenges such as limited curricular space and need for standardized training.
Roveta et al. (68)	Narrative review	General medical education	Adaptive learning, simulation, assessment, AI literacy	Concludes that AI can support adaptive learning, simulation, and objective assessment, but requires longitudinal validation, standardized benchmarks, and AI literacy for both learners and educators.
Jassar et al. (69)	Scoping review	Radiology education/competency development	Knowledge, skills, attitudes, competency domains	Proposes competency-based AI education in radiology, emphasizing ethics, bias, implementation, and regulation; useful for curriculum design but centered on competency mapping rather than intervention outcomes.
Negrete et al. (70)	Narrative review	Dent maxillofacial radiology education	Adaptive learning, feedback, competency-based learning, ethics	Shows that AI can enhance personalized learning, formative assessment, and competency-based dental radiology education, but requires infrastructure, faculty development, and ethical oversight.
Shaw et al. (71)	Scoping review	Medical education	Surgical skills, radiology training, interactive learning, text interpretation	Identifies radiology as one of four core AI application domains in medical education and concludes that evidence is promising but still limited and exploratory.
Hui et al. (72)	Scoping review	Radiology education	AI-assisted learning, diagnostic support, automated assessment	Finds that AI integration in radiology education is still emerging, with applications in curriculum generation, diagnostic support, and evaluation systems, but long-term evidence remains limited.
Sharma et al. (73)	Narrative review	Biomedical science curricula	AI literacy, curriculum reform, data science, ethics	Argues for integrating AI, informatics, and digital health into curricula to prepare future healthcare professionals, while noting challenges in standardization and implementation.
Tadavarthi et al. (74)	Qualitative/narrative review	Radiology practice and resident education	Workflow, quality, safety, reporting, education	Reviews noninterpretive AI applications in radiology, including resident education, customized teaching files, workflow optimization, and reporting support.
Yang et al. (75)	Scoping review	Stakeholder perspectives in radiology	Perceptions, trust, education, medicolegal implications	Reports that most stakeholders do not believe AI will replace radiologists, but strongly emphasize the need for education, training, and implementation guidance.
Iqbal et al. (76)	Systematic review	Healthcare during COVID-19	Public health prediction, radiology diagnosis, decision support	Shows that AI played a major role in radiology diagnosis, epidemiology, and decision support during the pandemic, though education was not its main focus.
Wade et al. (77)	Systematic review and meta-analysis	Undergraduate radiology education	Active vs. passive learning, eLearning vs. face-to-face, knowledge/skills	Shows that radiology teaching interventions generally improve learning; active learning trends favor better gains, and eLearning is at least comparable to face-to-face teaching, though heterogeneity is high.
Salehi et al. (78)	Systematic review	Medical imaging/AI technology	CNNs, transfer learning, classification, segmentation, challenges	Reviews technical advances in CNNs and transfer learning for medical imaging and highlights their promise but focuses on algorithms rather than educational implementation.
Zhang et al. (79)	Systematic review	Medical imaging/deep learning	CNNs, RNNs, GANs, segmentation, classification, reconstruction	Summarizes deep learning architectures and applications across imaging modalities, emphasizing diagnostic potential, data limitations, and interpretability challenges.
Huang et al. (80)	Systematic review	Medical imaging AI/classification	SSL strategies, unlabeled data, implementation guidance	Demonstrates that self-supervised learning can reduce dependence on labeled datasets and improve medical image classification, offering methodological guidance for future AI model development.
Present review	Structured review with scoping-oriented narrative synthesis	Radiology education across undergraduate medical education, residency training, and continuing professional development	Engagement, performance, and skill development in AI-supported educational interventions	The reviewed literature indicates growing interest in the use of artificial intelligence across radiology training environments. Reported educational applications included AI-assisted diagnostic software, AI literacy curricula, adaptive learning tools, and LLM-based educational support. Some publications reported favorable learner perceptions or early educational findings, although methodological limitations and heterogeneous outcome measures remain.

Numerous prior reviews have emphasized the need for AI literacy and curriculum reform within radiology and healthcare education. For example, a narrative review of radiology residency education highlighted the importance of structured AI curricula, ethical training, and clinical exposure to AI systems while identifying limited curricular space and the absence of standardized educational pathways as major barriers (67). Similarly, reviews addressing broader biomedical and medical education advocated the incorporation of AI literacy, digital health, informatics, and data science competencies into healthcare curricula to prepare future professionals for technologically enhanced clinical environments (73). The present review supports these recommendations but extends the discussion by demonstrating that the field has evolved beyond awareness-building and literacy-focused initiatives toward competency development, practical training, and performance-oriented educational interventions.

The current findings also reinforce and expand upon previous work exploring competency-based AI education. A recent scoping review proposed essential competency domains for radiology education, including technical knowledge, ethical awareness, implementation science, bias recognition, and regulatory understanding (69). Although that review provided a valuable framework for curriculum development, its primary objective was competency identification rather than evaluating how such competencies are developed through educational interventions. In contrast, our review synthesizes evidence from both educational and training-focused studies, allowing for a more comprehensive assessment of competency acquisition, educational outcomes, learner preparedness, and implementation challenges. This broader perspective enables the identification of relationships between educational strategies and reported outcomes that are not captured in competency-mapping exercises alone.

The present findings are likewise consistent with broader reviews of AI in medical education. Previous scoping reviews identified radiology as one of the principal domains in which AI has demonstrated educational utility through image interpretation support, interactive learning environments, and automated assessment systems (71). Similarly, reviews in dental and maxillofacial radiology education reported that AI can facilitate adaptive learning, personalized feedback, and competency-based instruction while emphasizing the need for faculty development and institutional support (70). Although these reviews provide important insights, they address either general medical education or highly specialized educational contexts. By focusing specifically on radiology education across undergraduate, postgraduate, residency, and professional training settings, the present review offers a more detailed and discipline-specific understanding of AI integration within imaging education.

Another notable contribution of this review is its examination of educational effectiveness and learner preparedness. Previous reviews have generally concluded that AI integration in radiology education remains in an early developmental stage and that evidence regarding long-term educational impact is limited (72). Similarly, stakeholder-focused reviews reported strong support for AI education while identifying concerns regarding implementation, trust, and professional adaptation (75). Our findings confirm these observations but additionally demonstrate that most educational interventions report positive or improved educational outcomes, suggesting that AI-focused educational initiatives can successfully enhance learner knowledge, confidence, and engagement. At the same time, the review highlights persistent deficiencies in preparedness, emphasizing that enthusiasm for AI frequently exceeds actual competency levels. Importantly, the present review captures emerging developments that are largely absent from earlier literature, particularly the growing educational role of generative AI and large language models. Recent studies evaluating these technologies have introduced new considerations related to information verification, ethical use, critical appraisal, and responsible implementation. These emerging themes reflect the rapidly changing nature of AI education and position the current review at the forefront of contemporary discussions regarding AI-enabled learning in radiology.

Finally, unlike several systematic reviews that focus primarily on technical advances in medical imaging AI—including deep learning architectures, convolutional neural networks, transfer learning, image classification, and segmentation methods (78–80)—the present review centers on educational implementation and workforce preparation. Rather than examining algorithm performance, it evaluates how learners, educators, and institutions engage with AI technologies and how educational systems can facilitate competency development. This distinction is particularly important because the successful integration of AI into healthcare depends not only on technological innovation but also on the preparedness of the professionals expected to use these systems responsibly and effectively. Taken together, the comparison with prior reviews demonstrates that the present study provides one of the most comprehensive syntheses currently available on AI in radiology education. By integrating evidence related to educational priorities, competency development, learner preparedness, educational effectiveness, training approaches, and institutional barriers across diverse educational contexts, this review advances the existing literature beyond isolated thematic analyses. So, it offers a more robust foundation for future curriculum development, competency framework design, educational policy, and research initiatives aimed at preparing the next generation of radiology professionals for an increasingly AI-enabled healthcare environment.

### 4.6 Limitations

While this scoping review provides a comprehensive overview of the current landscape of AI in radiology education, several limitations should be acknowledged when interpreting its findings. These limitations arise both from the characteristics of the available literature and from the inherent methodological nature of scoping reviews. The included studies demonstrated considerable heterogeneity across multiple dimensions, including educational settings, learner populations, intervention designs, study methodologies, outcome measures, and reporting practices. Educational initiatives ranged from surveys exploring perceptions and readiness to structured curricula, workshops, AI-assisted learning systems, and competency-development programs. Similarly, participants included medical students, radiology residents, radiographers, medical physicists, practicing clinicians, and other healthcare professionals. This diversity reflects the multidisciplinary and evolving nature of AI education but limits direct comparisons among studies and prevents the identification of universally applicable educational approaches.

Much of the available evidence relied on cross-sectional designs and self-reported outcomes. Measures such as perceived knowledge, confidence, attitudes, readiness, and satisfaction were frequently used to evaluate educational effectiveness. Although these indicators provide valuable insight into learner experiences and educational needs, they may not accurately reflect actual competency acquisition, objective performance, or the ability to apply AI knowledge in clinical practice. Consequently, the reported educational benefits should be interpreted with caution, as improvements in confidence do not necessarily translate into measurable professional competence. Another important limitation is the predominance of single-center studies and relatively small sample sizes. Many investigations were conducted within specific institutions or educational programs, potentially limiting the generalizability of their findings. Differences in institutional resources, technological infrastructure, faculty expertise, healthcare systems, and educational cultures may significantly influence the implementation and outcomes of AI-related educational initiatives. As a result, findings reported in one setting may not be directly transferable to other institutions or geographic regions.

The review also highlights a lack of longitudinal evidence. Most studies evaluated short-term educational outcomes immediately following an intervention or training activity, while relatively few assessed long-term knowledge retention, sustained competency development, behavioral changes, or the impact of AI education on clinical practice. This limitation restricts understanding of whether educational gains persist over time and whether they ultimately influence professional performance, decision-making, or patient care, there was substantial variability in how AI competencies were defined, taught, and assessed. The absence of standardized educational frameworks and validated assessment tools complicates comparisons across studies and hinders the establishment of best practices. This inconsistency reflects the developmental stage of the field and underscores the need for consensus regarding core competencies, learning objectives, and evaluation strategies.

Even with these limitations, this review provides a robust synthesis of current evidence and offers valuable insights into educational priorities, learner preparedness, training approaches, and implementation challenges. By identifying existing gaps and emerging trends, it establishes an important foundation for future research, curriculum development, and the advancement of competency-based AI education within radiology.

### 4.7 Integrating radiomics education into future radiology curricula

AI literacy should extend beyond machine learning algorithms and generative AI to include foundational knowledge of radiomics and quantitative imaging biomarkers (81). Radiomics extracts high-dimensional quantitative features from standard medical images to characterize tissue phenotype and has demonstrated potential for improving disease diagnosis, prognosis, treatment response assessment, and precision medicine (82). As radiomics becomes increasingly integrated into research and clinical decision-support systems, future radiologists will require a basic understanding of its principles and clinical applications (83). Educational programs should introduce the fundamental stages of the radiomics workflow, including image acquisition, segmentation, feature extraction, feature selection, model development, and validation (82, 83). Equally important is developing learners' ability to critically evaluate radiomics studies by recognizing common methodological challenges, such as variability in imaging protocols, limited reproducibility, overfitting, dataset bias, and insufficient external validation (84). Familiarity with standardization initiatives, including the Image Biomarker Standardisation Initiative (IBSI), may further strengthen trainees' capacity to assess the reliability and clinical applicability of radiomics-derived biomarkers (81, 83).

Rather than training radiologists to become radiomics developers, educational curricula should emphasize informed interpretation and critical appraisal. Integrating these competencies alongside broader AI literacy would better prepare future radiologists to collaborate effectively with data scientists, interpret quantitative imaging biomarkers within appropriate clinical contexts, and support the responsible implementation of AI-enabled precision imaging. As quantitative imaging technologies continue to evolve, incorporating radiomics education into radiology training represents an important step toward preparing the next generation of imaging specialists for increasingly data-driven clinical practice.

## 5 Conclusion

This scoping showed that skill development was the dominant educational priority, reflecting increasing efforts to equip learners with competencies related to machine learning, deep learning, algorithm interpretation, ethical considerations, clinical implementation, and critical appraisal of AI systems. Educational interventions, including structured curricula, workshops, lecture series, journal clubs, AI-assisted learning tools, and competency-based training initiatives, consistently reported improvements in learner knowledge, confidence, engagement, and preparedness. Also, the predominance of empirical studies indicates a progressive shift from conceptual discussions toward evidence-based evaluation of educational strategies and learner outcomes. In the face of these encouraging developments, the review identified a persistent gap between enthusiasm for AI and actual readiness for implementation. Across undergraduate students, residents, radiographers, medical physicists, and practicing professionals, learners generally recognized the importance of AI for future clinical practice but frequently reported limited formal training, insufficient confidence, and inadequate understanding of core AI concepts. This discrepancy suggests that current educational efforts remain insufficient to meet the growing demands of AI-integrated healthcare systems.

The findings also highlight several barriers that continue to hinder effective implementation of AI education in radiology. Limited faculty expertise, curriculum overload, inadequate institutional resources, lack of standardized competency frameworks, insufficient assessment strategies, and concerns regarding ethics, transparency, bias, privacy, and regulation were consistently reported across studies. The rapid evolution of AI technologies, particularly generative AI and large language models, further complicates curriculum development and underscores the need for flexible educational models capable of adapting to technological change. Inclusive, the evidence suggests that the future of AI education in radiology should move beyond introductory literacy toward structured, competency-based, and longitudinal educational frameworks. Successful programs will likely require integration across multiple stages of professional development, combining foundational knowledge, practical application, critical evaluation skills, ethical reasoning, and interdisciplinary collaboration. By addressing existing educational gaps and implementation challenges, radiology education can ensure that future professionals are prepared not only to use AI technologies effectively but also to evaluate, govern, and integrate them responsibly into clinical practice, AI education should be viewed as a strategic priority for radiology training programs seeking to prepare a workforce capable of thriving in an increasingly data-driven and technologically advanced healthcare environment.

## Data Availability

The original contributions presented in the study are included in the article/Supplementary Material, further inquiries can be directed to the corresponding authors.
