# Supplementary material for: Use of artificial intelligence in education and training of radiology
Source: Front Radiol. 2026 Sep 1;6:1899739. doi: 10.3389/fradi.2026.1899739 (PMC13574975; doi:10.3389/fradi.2026.1899739)
Supplement: Supplementary file 1 [file Datasheet1.docx]

**SUPPLEMENTARY INFORMATION: Use of Artificial Intelligence in Education and Training of Radiology**

María Belén Morales-Cevallos^1,^*, Canva Byron Ma Lam^2,^*, Diego Fabián Vique López^3^, Adriana Monserrath Monge Moreno^4^, María José López Pino^5^

^1^Universidad Ecotec, Km. 13.5 Samborondón, Samborondón, EC092302, Ecuador

^2^Universidad Católica Santiago de Guayaquil, Ecuador

^3^Italian University Institute of Rosario (IUNIR), Rosario, Argentina

^4^Facultad de Ciencias, Escuela Superior Politécnica de Chimborazo (ESPOCH), Riobamba 060155, Ecuador

^5^Facultad de Salud Pública, Escuela Superior Politécnica de Chimborazo (ESPOCH), Riobamba 060155, Ecuador

*** Correspondence:**
[mmoralesc@ecotec.edu.ec](mailto:mmoralesc@ecotec.edu.ec) (M.B.M-C) & [canva.ma@cu.ucsg.edu.ec](mailto:canva.ma@cu.ucsg.edu.ec) (C.B.M.L.)

**Table S1.** Complete search strategies used in Scopus, PubMed, and IEEE Xplore, including database-specific syntax, field tags, Boolean operators, and applied search filters.

| **SCOPUS** | TITLE-ABS-KEY  (  ("artificial intelligence" OR AI OR "machine learning"  OR "deep learning" OR "generative AI"  OR "large language model*" OR ChatGPT)  AND  (radiology OR radiologic OR radiologist* OR imaging)  AND  (education OR training OR curriculum  OR teaching OR learning OR student*  OR resident* OR educator*)  )  AND PUBYEAR > 2019  AND PUBYEAR < 2026  AND (LIMIT-TO(DOCTYPE,"ar"))  AND (LIMIT-TO(LANGUAGE,"English")) |
| --- | --- |
| **PubMed** | ("artificial intelligence"[Title/Abstract]  OR AI[Title/Abstract]  OR "machine learning"[Title/Abstract]  OR "deep learning"[Title/Abstract]  OR "generative AI"[Title/Abstract]  OR "large language model*"[Title/Abstract]  OR ChatGPT[Title/Abstract])  AND  (radiology[Title/Abstract]  OR radiologist*[Title/Abstract]  OR radiologic[Title/Abstract]  OR imaging[Title/Abstract])  AND  (education[Title/Abstract]  OR training[Title/Abstract]  OR curriculum[Title/Abstract]  OR teaching[Title/Abstract]  OR learning[Title/Abstract]  OR student*[Title/Abstract]  OR resident*[Title/Abstract]  OR educator*[Title/Abstract])  )  Filters:  Publication dates from 2020/01/01 to 2025/12/31;  English;  Journal Article. |
| **IEEE Xplore** | (  ("All Metadata":"artificial intelligence"  OR "All Metadata":"AI"  OR "All Metadata":"machine learning"  OR "All Metadata":"deep learning"  OR "All Metadata":"generative AI"  OR "All Metadata":"large language model"  OR "All Metadata":"ChatGPT")  AND  ("All Metadata":"radiology"  OR "All Metadata":"radiologist"  OR "All Metadata":"radiologic"  OR "All Metadata":"medical imaging")  AND  ("All Metadata":"education"  OR "All Metadata":"training"  OR "All Metadata":"curriculum"  OR "All Metadata":"teaching"  OR "All Metadata":"learning")  ) |

**Prompt Scheme 1**

Create a clean, modern medical infographic illustrating the integration of artificial intelligence into the radiology workflow. The scene should depict a contemporary radiology reading room with two healthcare professionals (a radiologist and a radiologic technologist or clinician) seated at a workstation surrounded by multiple high-resolution diagnostic monitors displaying anonymized brain MRI and CT scans with AI-generated overlays, lesion detection markers, heatmaps, segmentation contours, and analytical dashboards. The interface should appear professional and clinically realistic, using subtle blue, teal, and gray tones.

Surround the central workstation with four minimalist vector-style icons connected conceptually to the workflow:

- Integration into Workflow: a flowchart with interconnected nodes and arrows representing seamless AI integration into clinical practice.
- AI-Assisted Diagnosis: a clipboard with a magnifying glass and checkmark symbolizing AI-supported image interpretation.
- Decision Support: a checklist with a large green checkmark representing evidence-based clinical decision assistance.
- Performance Monitoring: a clock or dashboard icon representing continuous quality assurance, efficiency, and workflow optimization.

The monitors should include realistic AI visualization elements such as probability maps, automated lesion detection, quantitative measurements, confidence scores, and structured reporting suggestions. The healthcare professionals should appear engaged in collaborative decision-making, with one reviewing imaging findings and the other examining digital reports or a tablet.

Use a flat vector medical illustration style with smooth gradients, crisp outlines, balanced composition, soft shadows, and high visual clarity. Maintain a clean white background with subtle blue accents, avoiding clutter. The overall design should resemble a high-quality figure suitable for publication in a scientific review article or medical journal, emphasizing human–AI collaboration, clinical decision support, radiology workflow integration, and AI-enhanced diagnostic performance. Use a wide landscape aspect ratio (16:9), high resolution, professional infographic quality, with no logos or watermarks

**Prompt Scheme 2**

Create a high-quality medical education infographic illustrating competency-based artificial intelligence (AI) training in radiology. The central scene should depict a modern simulation laboratory where a multidisciplinary team of radiology trainees (medical students, radiology residents, and educators) interact with AI-enabled educational technologies. One learner is using a virtual reality (VR) headset to explore 3D medical anatomy, while others analyze interactive radiological images on a large digital touchscreen displaying CT, MRI, and ultrasound scans with AI-generated annotations, segmentation maps, confidence scores, and educational dashboards. A large monitor in the background should display a "Learner Preparedness Scorecard" with skill proficiency indicators, competency metrics, learning progress, and personalized recommendations.

Surround the central illustration with four professional vector-style educational panels:

Top Left – Simulation-Based Training & Practical Integration

- Show healthcare trainees practicing image interpretation in a simulated clinical environment.
- Include a simple competency workflow diagram with connected boxes labeled Skills → Practice → Assessment.
- Emphasize experiential learning and AI-assisted simulation.

Bottom Left – Objective Skills Assessment & Certification

- Display an OSCE-style examination scenario with an instructor evaluating a trainee.
- Include a clipboard with a checklist, certification badge, and completion checkmark representing competency-based assessment and credentialing.

Top Right – Peer Learning & Feedback Mechanisms

- Illustrate a collaborative discussion among radiology residents, clinicians, and faculty reviewing AI-generated imaging results.
- Include icons representing communication, feedback, mentoring, knowledge sharing, and collaborative learning.

Bottom Right – Longitudinal Performance Tracking

- Display a dashboard with progress charts, competency milestones, timelines, learning curves, and achievement markers representing continuous professional development and lifelong learning.

The overall composition should emphasize competency-based medical education (CBME), AI literacy, simulation-based learning, objective assessment, collaborative education, personalized learning analytics, and continuous competency monitoring.

Use a clean flat vector illustration style with subtle gradients, crisp outlines, soft shadows, and a professional medical color palette dominated by blue, teal, green, and white. Ensure a balanced layout with clear visual hierarchy, publication-quality resolution, minimal background clutter, and no logos or watermarks. The infographic should be suitable for inclusion in a peer-reviewed scientific review article on AI in radiology education, using a wide 16:9 landscape format

**Prompt Scheme 3**

Create a professional medical education infographic illustrating diverse AI-enabled teaching modalities in radiology education. Design the infographic in a clean 2 × 2 grid layout with a central arrow labeled "Diverse Educational Modalities" connecting the four educational approaches. Use a modern flat-vector medical illustration style with subtle gradients, clean outlines, soft shadows, and a blue–green healthcare color palette on a white background. The infographic should be publication quality, suitable for a peer-reviewed scientific review article.

Upper Left – High-Fidelity Simulation

- Illustrate a realistic simulation laboratory where healthcare trainees practice using a high-fidelity patient simulator.
- Display physiological monitors, AI-assisted patient monitoring dashboards, and medical imaging screens showing CT and MRI images.
- Include instructors supervising the simulation while learners perform clinical assessment and decision-making.
- Convey experiential learning, procedural training, and safe clinical practice.

Upper Right – E-Learning & Blended Modules

- Depict students participating in an online AI-enhanced radiology course using laptops and tablets.
- Show a virtual instructor on a large video-conferencing screen.
- Include digital checklists, learning management system dashboards, quizzes, progress tracking, and AI-powered personalized learning recommendations.
- Represent flexible, hybrid, and asynchronous learning environments.

Lower Left – Interactive Digital Learning

- Show learners collaboratively exploring interactive 3D anatomy, medical imaging, and AI-generated visualization dashboards on a large multitouch digital table.
- Display quantitative imaging analytics, segmentation overlays, organ models, performance charts, and adaptive learning feedback.
- Emphasize AI-assisted visualization, image interpretation, and self-directed learning.

Lower Right – Collaborative Problem-Solving

- Illustrate an interdisciplinary team of radiologists, residents, medical students, technologists, and clinicians collaboratively reviewing multimodal medical images on an interactive digital workstation.
- Display AI-generated annotations, lesion detection, structured reports, quantitative biomarkers, and discussion of diagnostic decisions.
- Emphasize teamwork, communication, clinical reasoning, and collaborative decision-making.

At the center of the infographic, include a large horizontal arrow labeled "Diverse Educational Modalities", designed with subtle circuit-board patterns to symbolize artificial intelligence and digital innovation. The arrow should visually connect all four educational strategies into one integrated competency-based educational ecosystem.

The overall figure should communicate AI-enhanced radiology education, emphasizing simulation-based learning, e-learning, interactive digital education, collaborative clinical reasoning, competency development, personalized learning, and technology-enhanced medical training. Maintain a clean scientific infographic appearance with balanced spacing, consistent typography, high visual clarity, 16:9 landscape orientation, and no logos, watermarks, or copyrighted elements.

**Prompt Scheme 4**

Create a publication-quality scientific infographic titled "Navigating the Pathway: AI Education in Radiology – Addressing Institutional Barriers Through Structured Pedagogy." Design a visually compelling conceptual roadmap using a modern flat-vector illustration style with subtle gradients, clean outlines, soft shadows, and a professional healthcare color palette of blue, teal, green, and gray. The infographic should have a wide 16:9 landscape layout suitable for inclusion in a peer-reviewed medical journal.

Overall Concept

Divide the infographic into two interconnected sections:

Upper section: Institutional barriers preventing effective implementation of AI education in radiology.

Lower section: Educational solutions demonstrating how structured pedagogy enables successful curriculum integration.

Connect both sections through a central transition pathway consisting of a broken bridge transforming into a bright educational roadmap, symbolizing the transition from barriers to sustainable educational implementation.

Header

Large professional title:

Navigating the Pathway: AI Education in Radiology

Subtitle:

Addressing Institutional Barriers Through Structured Pedagogy

Upper Section — Overcoming Institutional Barriers

Use a muted gray color palette to represent obstacles.

Create four illustrated institutional barriers:

1. Limited Faculty Expertise

Faculty members with limited AI knowledge.

Books, lectures, and outdated educational materials.

Broken graduation cap or incomplete knowledge icon.

Small labels:

Lack of AI/ML Training

Fragmented Instruction

Limited Faculty Development

2. Curricular Constraints

Maze representing overloaded curricula.

Educational calendar, books, and scheduling conflicts.

Labels:

Existing Learning Burden

Overcrowded Curriculum

Standalone Workshops Insufficient

3. Resource Limitations

Servers, computers, cloud infrastructure, and financial barriers.

Limited access to AI software and imaging datasets.

Labels:

Funding Limitations

Digital Infrastructure

Technology Access

4. Rapidly Evolving Technology

Clock, gears, software updates, regulatory documents.

AI chip with changing arrows.

Labels:

Continuous Technological Change

Regulatory Evolution

Outdated Educational Resources

Place these four barriers on elevated platforms separated by gaps.

Central Transition

Illustrate:

A broken bridge gradually transforming into a complete bridge.

Flowing river beneath labeled:

Educational Transformation

At the center place:

Strategic Reform

Subtitle:

Structured Transition Toward Sustainable AI Education

Multiple arrows should point upward toward the educational solutions.

Lower Left — Structured Pedagogy

Illustrate a modern medical school building connected with competency-based education.

Title:

Structured Pedagogy

Include icons for:

Learning objectives

Curriculum mapping

Competency frameworks

Progressive learning

Objective assessment

Continuous evaluation

Use green and blue colors indicating educational growth.

Lower Center — Enduring Competencies

Show an open library or educational platform with books labeled:

AI Literacy

Critical Appraisal

Ethical Reasoning

Algorithm Evaluation

Evidence-Based Implementation

Clinical Decision Support

Human–AI Collaboration

Data Governance

Add upward arrows indicating competency development.

Lower Right — Interdisciplinary Collaboration

Illustrate radiologists, educators, computer scientists, data scientists, engineers, and medical students collaborating around an interactive digital imaging workstation displaying:

CT

MRI

AI heatmaps

Segmentation overlays

Structured reports

Quantitative imaging biomarkers

Include labels:

Integrated Curriculum

Clinical Practice

AI Implementation

Collaborative Learning

Precision Education

Design Style

Professional scientific infographic.

Medical journal publication quality.

Flat vector illustration with subtle gradients.

Soft shadows.

Balanced composition.

Clean typography.

High resolution.

White background.

Blue, teal, green, and gray professional medical palette.

No logos.

No watermarks.

No copyrighted elements.

Highly polished visual suitable for a review article on artificial intelligence in radiology education.
